# *Circ-HuR* suppresses HuR expression and gastric cancer progression by inhibiting CNBP transactivation

**DOI:** 10.1186/s12943-019-1094-z

**Published:** 2019-11-13

**Authors:** Feng Yang, Anpei Hu, Dan Li, Jianqun Wang, Yanhua Guo, Yang Liu, Hongjun Li, Yajun Chen, Xiaojing Wang, Kai Huang, Liduan Zheng, Qiangsong Tong

**Affiliations:** 1 Department of Pediatric Surgery, Union Hospital, Tongji Medical College, Huazhong University of Science and Technology, 1277 Jiefang Avenue, Wuhan, Hubei Province 430022 People’s Republic of China; 2 Department of Pathology, Union Hospital, Tongji Medical College, Huazhong University of Science and Technology, 1277 Jiefang Avenue, Wuhan, Hubei Province 430022 People’s Republic of China; 3 Clinical Center of Human Genomic Research, Union Hospital, Tongji Medical College, Huazhong University of Science and Technology, 1277 Jiefang Avenue, Wuhan, Hubei Province 430022 People’s Republic of China

**Keywords:** Circular RNAs, Human antigen R, Gastric cancer, CCHC-type zinc finger nucleic acid binding protein

## Abstract

**Background:**

Circular RNAs (circRNAs), a subclass of non-coding RNAs, play essential roles in tumorigenesis and aggressiveness. Our previous study has identified that *circAGO2* drives gastric cancer progression through activating human antigen R (HuR), a protein stabilizing AU-rich element-containing mRNAs. However, the functions and underlying mechanisms of circRNAs derived from *HuR* in gastric cancer progression remain elusive.

**Methods:**

CircRNAs derived from *HuR* were detected by real-time quantitative RT-PCR and validated by Sanger sequencing. Biotin-labeled RNA pull-down, mass spectrometry, RNA immunoprecipitation, RNA electrophoretic mobility shift, and in vitro binding assays were applied to identify proteins interacting with circRNA. Gene expression regulation was observed by chromatin immunoprecipitation, dual-luciferase assay, real-time quantitative RT-PCR, and western blot assays. Gain- and loss-of-function studies were performed to observe the impacts of circRNA and its protein partner on the growth, invasion, and metastasis of gastric cancer cells in vitro and in vivo*.*

**Results:**

*Circ-HuR* (*hsa_circ_0049027*) was predominantly detected in the nucleus, and was down-regulated in gastric cancer tissues and cell lines. Ectopic expression of *circ-HuR* suppressed the growth, invasion, and metastasis of gastric cancer cells in vitro and in vivo. Mechanistically, *circ-HuR* interacted with CCHC-type zinc finger nucleic acid binding protein (CNBP), and subsequently restrained its binding to *HuR* promoter, resulting in down-regulation of *HuR* and repression of tumor progression.

**Conclusions:**

*Circ-HuR* serves as a tumor suppressor to inhibit CNBP-facilitated *HuR* expression and gastric cancer progression, indicating a potential therapeutic target for gastric cancer.

## Background

Gastric cancer is one of the leading causes of cancer-related death, mainly due to high rate of recurrence and distant metastasis in advanced cases [1]. Thus, it is urgent to elucidate the mechanisms underlying the progression of gastric cancer. Human antigen R (HuR) is a member of the embryonic lethal abnormal visual protein (ELAV) family that is involved in nervous system development, cellular proliferation, and migration [2]. As a RNA binding protein (RBP), HuR increases mRNA stability of target genes through binding to poly-U elements or AU-rich elements (AREs) in 3′-untranslated region (3′-UTR) [3], and plays an important role in tumor development, recurrence, invasion, and metastasis [4]. Studies have shown that HuR is highly expressed in breast cancer, colorectal cancer, gastric cancer, and prostate cancer, and is closely related to clinicopathological features, lymph node metastasis, low survival rate, and poor prognosis of cancer patients [5–9]. However, the mechanisms regulating *HuR* expression during gastric cancer progression still remain to be elucidated.

Circular RNAs (circRNAs), a group of transcripts characterized by closed continuous loops stably existing in tissues and cells, are essential regulators of gene expression, while dysregulated circRNAs have been identified in almost all types of cancers [10]. Previous studies show that circRNAs play multiple important roles in cellular physiology via acting as microRNA (miRNA) sponges, RBP-binding molecules, transcriptional regulators, or templates for protein translation. The inspiring miRNA sponging activity has been proven in *CDR1as* and *ciRS-7* [11, 12], and *ciRS-7* contains 70 target sites of miRNA-7 to act as a competing endogenous RNA. However, the majority of circRNAs harbor few binding sites for a single miRNA, and high-throughput sequencing analysis reveals that miRNA sponging mechanism cannot be widely applied across the properties of circRNAs [13]. Recent studies have shown the emerging roles of circRNAs in control of gene expression via physical interaction with proteins. For example, *circ-Amotl1* derived from angiomotin-like 1 (*AMOTL1*) promotes tumorigenesis by binding to and retaining c-Myc within the nuclei in breast cancer [14]. *Circ-CTNNB1* generated by β-catenin (*CTNNB1*) activates Wnt signaling pathway by interacting with DEAD-box polypeptide 3 [15]. In addition, *circ-EIF3J* generated by eukaryotic translation elongation factor 3 J (*EIF3J*) interacts with U1 snRNP and RNA polymerase II to promote parental gene transcription [16]. In our previous work, we have demonstrated that HuR is highly expressed in gastric cancer tissues and cells, while circRNA derived from Argonaute 2 (*circAGO2*) binds and promotes the recruitment of HuR protein to the 3′-UTR of target genes, and facilitates the proliferation, invasion, and metastasis of gastric cancer cells, suggesting that circRNA can regulate HuR activity in gastric cancer [17]. Meanwhile, the roles of circRNAs in regulating *HuR* expression in gastric cancer still remain largely elusive.

In this study, we identify a circRNA consisting of exons 3, 4, and 5 of *HuR* (*circ-HuR*) as a novel tumor suppressor in gastric cancer. We discover that *circ-HuR* is significantly down-regulated in gastric cancer, and effectively inhibits the growth, invasion, and metastasis of gastric cancer cells. Mechanistically, *circ-HuR* directly interacted with CCHC-type zinc finger nucleic acid binding protein (CNBP), and acted as an inhibitor to restrain the binding of CNBP to *HuR* promoter, resulting in repression of *HuR* expression and tumor progression, which indicates the essential roles of *circ-HuR* and *CNBP* in gastric cancer progression.

## Materials and methods

### Patient tissues and cell culture

Tumor and adjacent normal (peritumor) tissues of 81 gastric cancer cases were obtained at surgery in Union Hospital of Tongji Medical College, with demographic and clinicopathological details indicated in Additional file 1: Table S1. The Institutional Review Board of Tongji Medical College approved human tissue study (approval number: 2011-S085). All procedures were carried out in accordance with guidelines set forth by Declaration of Helsinki. Written informed consent was obtained from all patients. Fresh tumor tissues were validated by pathological diagnosis, frozen in liquid nitrogen, and stored at − 80 °C. Human embryonic kidney HEK293T (CRL-11268) cells and gastric cancer cell lines AGS (CRL-1739), MKN-45 (JCRB0254), MKN-74 (JCRB0255) and NCI-N87 (CRL-5822) were obtained from American Type Culture Collection (Rockville, MD) and Japanese Collection of Research Bioresources Cell Bank (Osaka, Japan), authenticated by short tandem repeat (STR) profiling, and applied for study within 6 months following resuscitation of frozen aliquots. Cell lines were cultured in RPMI 1640 medium (Gibco, Carlsbad, CA, USA) supplied with 10% fetal bovine serum (Gibco) at 37 °C in a humidified atmosphere of 5% CO_2_.

### RT-PCR and real-time quantitative RT-PCR (qRT-PCR)

Total RNA was isolated according to the instructions of RNeasy Mini Kit (QIAGEN, Stockach, Germany). For circRNA detection, treatment with RNase R (3 U/mg, Epicenter, Madison, WI, USA) was undertaken at 37 °C for 15 min, and cDNA was synthesized by using reverse transcription kit (Takara, Dalian, China). Genomic DNA (gDNA) was isolated with DNA Mini Kit (QIAGEN). Quantification of mRNA, circular RNA and gDNA was performed by using a SYBR Green PCR Kit (Takara), primers (Additional file 1: Table S2) and Real-Time PCR System (Applied Biosystems, Carlsbad, CA, USA). The levels of circRNA and mRNA were normalized to those of β-actin and determined by 2^-△△Ct^ method.

### Western blot assay

Cellular proteins were extracted with RIPA lysis buffer (Thermo Fisher Scientific, Inc., Waltham, MA, USA). Western blot assay was performed as previously described [18, 19], with antibodies specific for HuR (ab200342), cyclin D2 (CCND2, ab207604), CTNNB1 (ab32572), CNBP (ab48027), enolase 1 (ENO1, ab155102), nucleosome assembly protein 1 like 1 (NAP1L1, ab33076), matrix metalloproteinases 14 (MMP-14, ab51074), c-Myc (ab32072), Flag (ab45766), β-actin (ab125402, Abcam lnc., Cambridge, MA, USA), heparin binding growth factor (HDGF, A10435), non-POU domain containing octamer binding (NONO, A5282), splicing factor proline and glutamine rich (SFPQ, A0958, ABclonal, Wuhan, China), glutathione S-transferase (GST, sc-33,614), histone H3 (sc-24,516), or glyceraldehyde 3-phosphate dehydrogenase (GAPDH, sc-47,724, Santa Cruz Biotechnology, Santa Cruz, CA, USA).

### Plasmid construction and stable transfection

Linear *circ-HuR* and *hsa_circ_23897* were synthesized by TSINGKE (Wuhan, China) and inserted into pLCDH-ciR (Geenseed Biotech Co., Guangzhou, China). Mutation of back-splicing elements of *circ-HuR* or *hsa_circ_23897* vector was prepared with GeneTailor™ Site-Directed Mutagenesis System (Invitrogen, Carlsbad, CA, USA) and primers (Additional file 1: Table S3). Human *CNBP* cDNA (540 bp) was subcloned into CV186 (Genechem Co., Ltd., Shanghai, China), while its truncations were obtained by PCR amplification (Additional file 1: Table S3) and inserted into pCMV-3Tag-1A or pGEX-6P-1 (Addgene, Cambridge, MA, USA), respectively. Two independent single guide RNAs (sgRNAs) targeting downstream region of *CNBP* transcription start site (Additional file 1: Table S4) were inserted into dCas9-BFP-KRAB (Addgene). Oligonucleotides specific for short hairpin RNAs (shRNAs) against *circ-HuR* (Additional file 1: Table S4) were inserted into GV298 (Genechem Co., Ltd). Lentiviral vectors were co-transfected with packaging plasmids psPAX2 and pMD2G into HEK293T cells. Infectious lentiviruses were harvested at 36 and 60 h after transfection, followed with concentration by ultracentrifugation (2 h at 120,000 g). Stable cell lines were obtained by selection with puromycin.

### RNA fluorescence in situ hybridization (RNA-FISH)

Digoxin (DIG)-labeled antisense or sense probe for *circ-HuR* junction sequence (Additional file 1: Table S4) was synthesized. The probes for *GAPDH* and *U1* were generated by in vitro transcription of PCR products (Additional file 1: Table S2) using DIG Labeling Kit (MyLab Corporation, Beijing, China). Hybridization was undertaken using Fluorescent In Situ Hybridization kit (RiboBio, Guangzhou, China) following the manufacturer’s instructions, while the nuclei were counterstained with 4′,6-diamidino-2-phenylindole (DAPI). The images were analyzed via a Nikon A1Si Laser Scanning Confocal Microscope (Nikon Instruments Inc., Japan).

### Dual-luciferase reporter assay

Human *HuR* promoter (1341 bp) or *c-Myc* promoter (1363 bp) was amplified from gDNA using primer sets (Additional file 1: Table S3) and subcloned into pGL3-Basic (Promega, Madison, WI, USA). Human *MMP-14* promoter reporter was previously described [20]. Mutation of CNBP binding site was established using GeneTailor™ Site-Directed Mutagenesis System (Invitrogen) and primers (Additional file 1: Table S3). Human CNBP activity luciferase reporter was established by inserting oligonucleotides containing four canonical CNBP binding sites (Additional file 1: Table S3) into pGL3-Basic (Promega). Dual-luciferase assay was performed according to the manufacturer’s instructions (Promega).

### Biotin-labeled RNA pull-down and mass spectrometry

Biotin-labeled oligonucleotide probes (Additional file 1: Table S4) targeting junction sites of circRNAs were synthesized (Invitrogen). Using Biotin RNA Labeling Mix kit (Roche, Indianapolis, IN, USA) and T7 RNA polymerase, the biotin-labeled RNA probes for circRNAs were in vitro transcribed as previously described [15, 17]. RNA pull-down assay was performed at room temperature, and retrieved proteins were detected by mass spectrometry analysis at Wuhan Institute of Biotechnology (Wuhan, China).

### Fluorescence immunocytochemical staining

Cells were plated on coverslip and incubated with antibody specific for CNBP (ab48027, Abcam lnc.) at 4 °C overnight, and incubated with Alexa Fluor 594 goat anti-rabbit IgG and DAPI. The images were photographed under a Nikon A1Si Laser Scanning Confocal Microscope (Nikon Instruments Inc., Japan).

### Chromatin immunoprecipitation (ChIP)

ChIP assay was undertaken in accordance with the manual of EZ-ChIP kit (Upstate Biotechnology, Temacula, CA, USA), with antibodies specific for CNBP (ab48027, Abcam lnc.). Primer sets were listed in Additional file 1: Table S2.

### Cross-linking RNA immunoprecipitation (RIP) assay

Cells were cross-linked at 254 nm by ultraviolet light, and RIP assay was performed according to the instructions of Magna RIPTM Kit (Millipore, Bedford, MA, USA), with antibodies specific for CNBP (ab48027), ENO1 (ab155102), NAP1L1 (ab33076), Flag (ab45766, Abcam lnc.), HDGF (A10435), NONO (A5282), SFPQ (A0958, ABclonal). Co-precipitated RNAs were detected by RT-PCR or real-time qRT-PCR with specific primers (Additional file 1: Table S2).

### In vitro binding assay

Four truncations of *CNBP* were amplified with primers (Additional file 1: Table S3), and subcloned into pCMV-3Tag-1A or pGEX-6P-1 (Addgene). GST-tagged CNBP protein was produced from *E. coli* as previously described [18, 21]. The Flag-CNBP, GST-CNBP, and *circ-HuR* complexes were pulled down using GST or Flag beads (Sigma, St. Louis, MO, USA). *Circ-HuR* was measured by RT-PCR with divergent primers (Additional file 1: Table S2), whereas protein was detected by SDS-PAGE and western blot.

### RNA electrophoretic mobility shift assay (EMSA)

Biotin-labeled circular probe of *circ-HuR* was prepared as described above. RNA EMSA was conducted according to the instructions of LightShift Chemiluminescent RNA EMSA Kit (Thermo Fisher Scientific, Inc.)

### In vitro cell viability, growth, and invasion assays

The in vitro viability, growth, and invasive capabilities of cancer cells were detected by MTT colorimetric, soft agar, and matrigel invasion assays as described previously [15, 21].

### Xenografts in nude mice

All animal experiments were carried out in accordance with NIH Guidelines for the Care and Use of Laboratory Animals, and approved by the Animal Care Committee of Tongji Medical College (approval number: Y20080290). In vivo tumor growth and experimental metastasis studies were performed with blindly randomized four-week-old male BALB/c nude mice (*n* = 5 per group). For tumor growth studies, AGS cells (1 × 10^6^) stably transfected with vectors were injected into the upper back of nude mice (*n* = 5 per group). For metastasis studies, AGS cells (0.4 × 10^6^) stably transfected with vectors were injected from tail vein of nude mice (*n* = 5 per group). The tumor volume and survival time of each mouse were monitored and recorded, while xenografts were imaged using In-Vivo Xtreme II small animal imaging system (Bruker Corporation, Billerica, MA) and detected by hematoxylin and eosin (H&E) and immunohistochemical staining.

### Immunohistochemistry

Immunohistochemical staining and quantitative evaluation was performed as previously described [15, 21], with antibodies specific for Ki-67 (sc-23,900, Santa Cruz Biotechnology; 1:100 dilution) or CD31 (ab28364, Abcam Inc.; 1:100 dilution). The degree of positivity was measured according to percentage of positive cancer cells.

### Statistical analysis

All data were shown as mean ± standard error of the mean (SEM) processed by GraphPad Prism 5.0 (La Jolla, USA). Student’s *t*-test, analysis of variance (ANOVA), and chi-square analysis were used to evaluate the difference. Pearson’s correlation coefficient assay was used to analyze expression correlation. Log-rank test and Cox regression models were used to assess survival difference and hazard ratio. All statistical tests were two-sided and considered statistically significant when *P* values less than 0.05.

## Results

### *Circ-HuR* is down-regulated and decreases *HuR* expression in gastric cancer

**Fig. 1:**
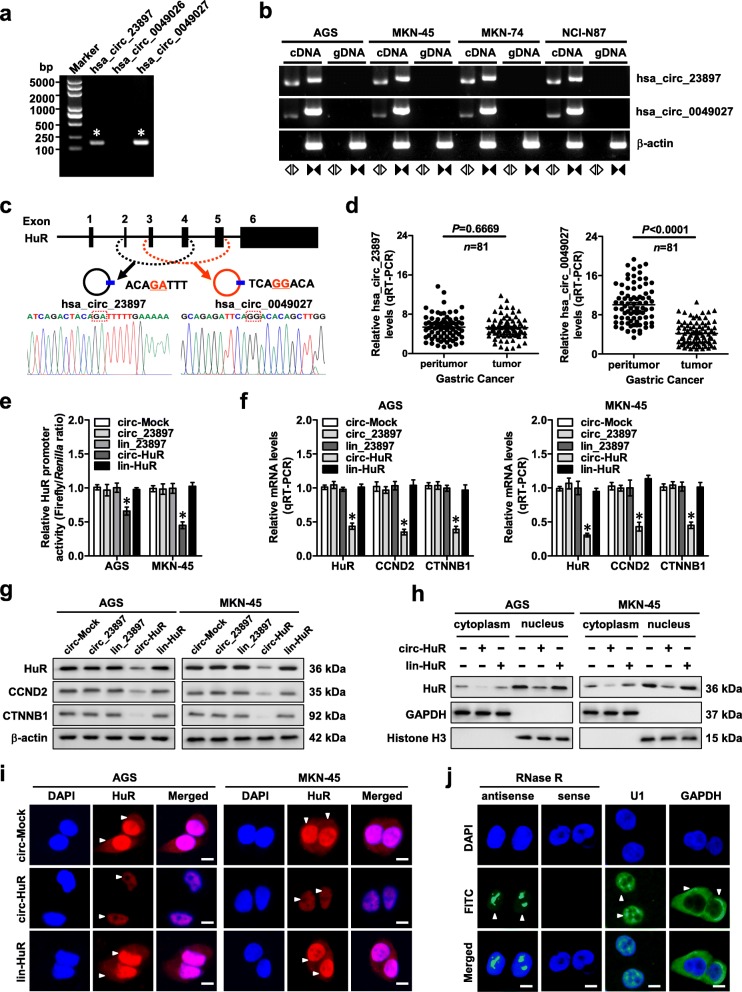
*Circ-HuR* is down-regulated and decreases *HuR* expression in gastric cancer. **a** RT-PCR assay with divergent primers indicating the detection of three circRNAs derived from *HuR* in AGS cells. **b** PCR assay with divergent and convergent primers showing the amplification of circRNAs from cDNA or genomic DNA (gDNA) of gastric cancer cell lines, while β-actin was used as a negative control. **c** Schematic illustration indicating the generation of *hsa_circ_23897* and *hsa_circ_0049027* from its host gene, and validation by Sanger sequencing. **d** Real-time qRT-PCR assay showing the relative levels (normalized to β-actin) of *hsa_circ_23897* or *hsa_circ_0049027* in the peritumor and tumor tissues of gastric cancer (*n* = 81). **e** Dual-luciferase assay indicating the promoter activity of *HuR* in AGS and MKN-45 cells stably transfected with empty vector (*circ-Mock*), *hsa_circ-23897* (*circ_23897*), linear *circ-23897* (*lin_23897*), *circ-HuR*, or linear *circ-HuR* (*lin-HuR*). **f** and **g** Real-time qRT-PCR (**f**, normalized to β-actin, *n* = 5) and western blot (**g**) assays revealing the transcript and protein levels of *HuR* and its downstream target genes in AGS and MKN-45 cells stably transfected with *circ-Mock*, *circ-23897*, *lin_23897*, *circ-HuR*, or *lin-HuR*. **h** and **i** Western blot (**h**) and immunofluorescence (**i**) assays showing the cytoplasmic and nuclear accumulation of HuR in AGS and MKN-45 cells stably transfected with *circ-Mock*, *circ-HuR*, or *lin-HuR*. Nuclei were stained by DAPI (blue). Scale bar, 10 μm. **j** RNA-FISH assay indicating the nuclear localization of *circ-HuR* in AGS cells using an antisense probe (green), while sense probe was used as a negative control. *U1* and *GAPDH* were applied as positive controls. Scale bar, 10 μm. Student’s *t*-test and ANOVA analyzed the difference in (**d**-**f**). *P < 0.01 vs. circ-Mock. Data are shown as mean ± SEM (error bars) and representative of three independent experiments in (**a**-**c**) and (**e**-**j**)

Analysis of circRNA sequencing databases circBase (http://www.circbase.org/) and circRNADb (http://reprod.njmu.edu.cn/circrnadb) revealed 3 potential circRNAs derived from *HuR*. Among them, *hsa_circ_23897* and *hsa_circ_0049027* were validated by PCR amplification using divergent primers from cDNA, but not from genomic DNA, of gastric cancer cell lines (Fig. 1a, b). *Hsa_circ_23897* was consisted of exons 2, 3, and 4 (446 nt), while *hsa_circ_0049027* was composed of exons 3, 4, and 5 of *HuR* (484 nt), and both of them were validated by Sanger sequencing (Fig. 1c). Endogenous *hsa_circ_23897* and *hsa_circ_0049027* were resistant to RNase R digestion, while linear mRNA of *HuR* in AGS and MKN-45 cells was significantly reduced by RNase R treatment (Additional file 1: Figure S1a). In gastric tissues and cell lines, *hsa_circ_0049027* was remarkably down-regulated, while *hsa_circ_23897* levels were not significantly altered (Fig. 1d and Additional file 1: Figure S1b). To investigate the functions of these circRNAs, *hsa_circ_23897* (*circ_23897*), *hsa_circ_0049027* (termed as *circ-HuR*), and their corresponding linear transcript (*lin_23897* and *lin-HuR*) constructs were stably transfected into AGS and MKN-45 cells (Additional file 1: Figure S1c). Ectopic expression of *circ-HuR*, but not of *circ_23897*, *lin_23897*, or *lin-HuR*, attenuated the promoter activity of *HuR* (Fig. 1e) and decreased the transcript and protein levels of *HuR* and its downstream genes *CCND2* [22] and *CTNNB1* [23] in AGS and MKN-45 cells (Fig. 1f, g). Meanwhile, stable transfection of two shRNAs targeting the junction of *circ-HuR* resulted in its down-regulation (Additional file 1: Figure S1d) and up-regulation of *HuR* and its downstream genes (*CCND2* and *CTNNB1*) in gastric cancer cells (Additional file 1: Figure S1e, f). Accordingly, ectopic expression of *circ-HuR*, rather than *lin-HuR*, attenuated the *HuR* expression in both nucleus and cytoplasm of gastric cancer cells (Fig. 1h, i). RNA-FISH assay indicated the endogenous nuclear enrichment of *circ-HuR* in AGS cells (Fig. 1j). Additionally, subcellular fractionation assay showed the nuclear localization of exogenous *circ-HuR* in AGS and MKN-45 cells stably transfected with *circ-HuR* (Additional file 1: Figure S1g). As a RBP involved in post-transcriptional control of gene expression, the mRNA-stabilizing function of HuR is linked to its localization in the cytoplasm [24, 25]. Neither ectopic expression nor knockdown of *circ-HuR* affected the half-life of *HuR* mRNA (Additional file 1: Figure S2a), whereas over-expression of *circ-HuR* decreased the half-life and transcript levels of *CCND2* and *CTNNB1* in a HuR-dependent manner (Additional file 1: Figure S2b, c). These results indicated that *circ-HuR* was down-regulated and suppressed the expression of *HuR* in gastric cancer.

### Over-expression of *circ-HuR* suppresses the growth and aggressiveness of gastric cancer

**Fig. 2:**
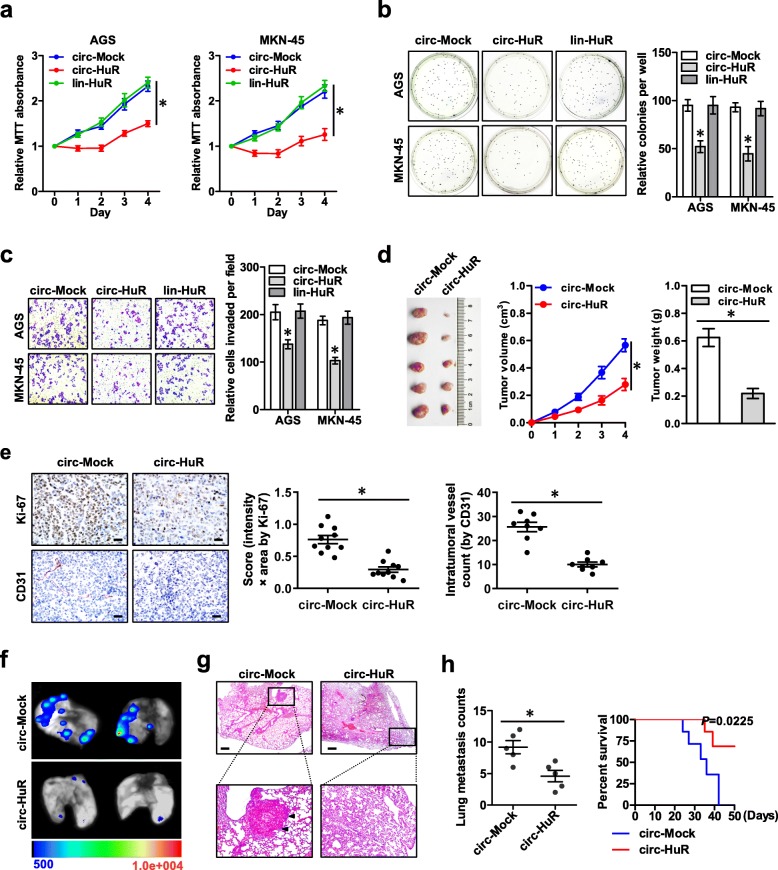
Over-expression of *circ-HuR* suppresses the growth and aggressiveness of gastric cancer. **a** MTT colorimetric assay showing the viability of AGS and MKN-45 cells stably transfected with empty vector (*circ-Mock*), *circ-HuR*, or *lin-HuR*. **b** and **c** Soft agar (**b**) and matrigel invasion (**c**) assays indicating the in vitro growth and invasion of AGS and MKN-45 cells stably transfected with *circ-Mock*, *circ-HuR*, or *lin-HuR*. **d** Representative (left panel), in vivo growth curve (middle panel), and weight at the end points (right panel) of xenograft tumors formed by subcutaneous injection of AGS cells stably transfected with *circ-Mock* or *circ-HuR* into the dorsal flanks of nude mice (*n* = 5 for each group). **e** Representative images (left panel) and quantification (right panel) of immunohistochemical staining showing the expression of Ki-67 and CD31 within xenograft tumors formed by hypodermic injection of AGS cells stably transfected with *circ-Mock* or *circ-HuR* (*n* = 5 for each group). Scale bars: 50 μm. **f**-**h** Representative images (**f**), H&E staining (**g**, arrowheads), and quantification (**h**, left panel) of lung metastatic colonization and Kaplan-Meier curves (**h**, right panel) of nude mice treated with tail vein injection of AGS cells stably transfected with mock or *circ-HuR* (*n* = 5 for each group). Scale bar: 100 μm. ANOVA and Student’s *t*-test analyzed the difference in **a**-**e** and **h**. Log-rank test for survival comparison in (**h**). *P < 0.01 vs. circ-Mock. Data are shown as mean ± SEM (error bars) and representative of three independent experiments in (**a**-**c**)

To explore the functions of *circ-HuR* in gastric cancer, its impacts on tumorigenesis and aggressiveness were investigated in AGS and MKN-45 cells with stable transfection of *circ-HuR* or its linear transcript (*lin-HuR*). In MTT colorimetric assay, stable ectopic expression of *circ-HuR*, but not of *lin-HuR*, decreased the viability of gastric cancer cells, than those stably transfected with empty vector (*circ-Mock*) (Fig. 2a). In soft agar and matrigel invasion assays, stable over-expression of *circ-HuR* reduced the growth and invasion capability of AGS and MKN-45 cells, respectively (Fig. 2b-c), while ectopic expression of *lin-HuR* did not affect these biological features of cancer cells (Fig. 2b-c). Consistently, stably transfection of *circ-HuR* resulted in a significant decrease in the growth and weight of xenograft tumors formed by subcutaneous injection of AGS cells into athymic nude mice (Fig. 2d). Immunohistochemical staining showed lower Ki-67 proliferation index and less CD31-positive microvessels of xenograft tumors formed by AGS cells stably transfected with *circ-HuR* (Fig. 2e). Importantly, athymic nude mice treated with tail vein injection of AGS cells stably transfected with *circ-HuR* displayed less lung metastatic colonies and greater survival probability (Fig. 2f-h). These results suggested that over-expression of *circ-HuR* suppressed the growth and aggressiveness of gastric cancer in vitro and in vivo.

### *Circ-HuR* interacts with CNBP protein in gastric cancer cells

**Fig. 3:**
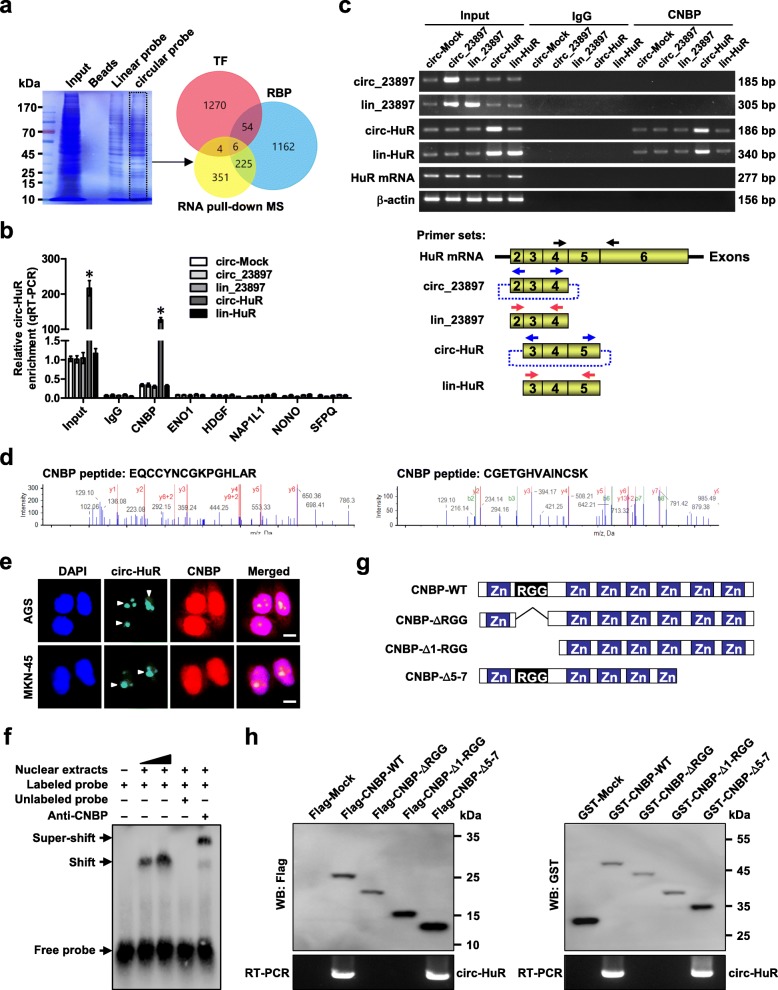
*Circ-HuR* interacts with CNBP protein in gastric cancer. **a** Coomassie bright blue staining (left panel), mass spectrometry (MS) assay, and overlapping analysis (Venn diagram, right panel) with established RBP and TF databases revealing the proteins pulled down by biotin-labeled linear or circular forms of *circ-HuR* from the lysates of AGS cells. **b** RIP and real-time qRT-PCR assays showing the relative interaction between *circ-HuR* and six proteins in AGS cells stably transfected with empty vector (*circ-Mock*), *hsa_circ-23897* (*circ_23897*), linear *circ-23897* (*lin_23897*), *circ-HuR*, or linear *circ-HuR* (*lin-HuR*), with normalization to input of cells transfected with *circ-Mock*. **c** RIP assay with primer sets (lower panel) indicating the interaction between *circ-HuR* and CNBP in AGS cells stably transfected with *circ-Mock*, *circ_23897*, *lin_23897*, *circ-HuR*, or *lin-HuR* (upper panel). **d** MS assay depicting the identified CNBP peptides pulled down by *circ-HuR*. **e** Dual RNA-FISH and immunofluorescence staining assay indicating the co-localization of *circ-HuR* (green) and CNBP (red) in AGS and MKN-45 cells, with nuclei staining with DAPI (blue). Scale bar, 5 μm. **f** RNA EMSA determining the interaction between endogenous CNBP protein and biotin-labeled circular probe of *circ-HuR* (arrowheads), with CNBP antibody incubation or competition using an excess of unlabeled circular probe of *circ-HuR*. **g** Schematic diagram revealing the domains of CNBP truncations. **h** In vitro binding assay showing the enriched *circ-HuR* levels detected by RT-PCR (lower panel) after incubation with full-length or truncations of Flag-tagged or GST-tagged recombinant CNBP protein validated by western blot (upper panel). ANOVA analyzed the difference in (**b**). *P < 0.01 vs. circ-Mock. Data are shown as mean ± SEM (error bars) and representative of three independent experiments in (**b**, **c**, **e**, **f**, and **h**)

To identify potential protein partner of *circ-HuR*, we performed proteomic analysis of *circ-HuR*-associated protein complex in AGS cells by biotin-labeled circular or linear RNA pull-down assay. The circular probe was gained by ligation of linear *circ-HuR* transcript in vitro and RNase R digestion (Additional file 1: Figure S3a). Mass spectrometry (MS) assay revealed 586 differential proteins between circular and linear *circ-HuR* pull-down groups, and overlapping analysis with established RBPs (http://www.ablife.cc) and transcription factors (TFs, http://www.genomatix.de) indicated six potential *circ-HuR*-interacting partners (Fig. 3a and Additional file 2: Table S5). By using probes generated by ligation of linear transcript or synthesized as oligonucleotides targeting junction site, further validating RNA pull-down assay indicated the interaction of *circ-HuR* with CNBP, rather than ENO1, HDGF, NAP1L1, NONO, or SFPQ, which was increased by over-expression of *circ-HuR* (Additional file 1: Figure S3b, c). Meanwhile, there was no interaction of CNBP with *lin-HuR* or *circ_23897* in AGS cells (Additional file 1: Figure S3b, c). RIP assay also indicated the binding of *circ-HuR* to CNBP, rather than ENO1, HDGF, NAP1L1, NONO, or SFPQ (Fig. 3b). Moreover, transfection of *circ-HuR*, but not of *lin-HuR*, *circ_23897*, or *lin_23897*, led to its increased enrichment in RNA co-precipitated by CNBP antibody in AGS cells (Fig. 3b, c and Additional file 1: Figure S3d). The identified peptides of CNBP from MS assay were shown in Fig. 3d. Dual RNA-FISH and immunofluorescence assay confirmed the co-localization of *circ-HuR* and CNBP in AGS and MKN-45 cells (Fig. 3e). RNA EMSA using cyclized probes showed that *circ-HuR* interacted with endogenous CNBP in the nuclear extracts of AGS cells (Fig. 3f). In vitro binding assay indicated that Arg-Gly-Gly (RGG) box domain (22–42 amino acids), but not other domains, of Flag-tagged or GST-tagged CNBP protein was crucial for its interaction with *circ-HuR* (Fig. 3g, h). Through bioinformatics analysis using UCSC Genome Browser (http://genome.ucsc.edu/), we found the CNBP binding motif (5′-AATGAGA-3′) [26] within *HuR* promoter, implicating the roles of CNBP in regulating *HuR* expression. These results suggested that *circ-HuR* interacted with CNBP, a protein with features of both RBP and TF, in gastric cancer cells.

### CNBP promotes *HuR* expression, growth, and aggressiveness of gastric cancer

**Fig. 4:**
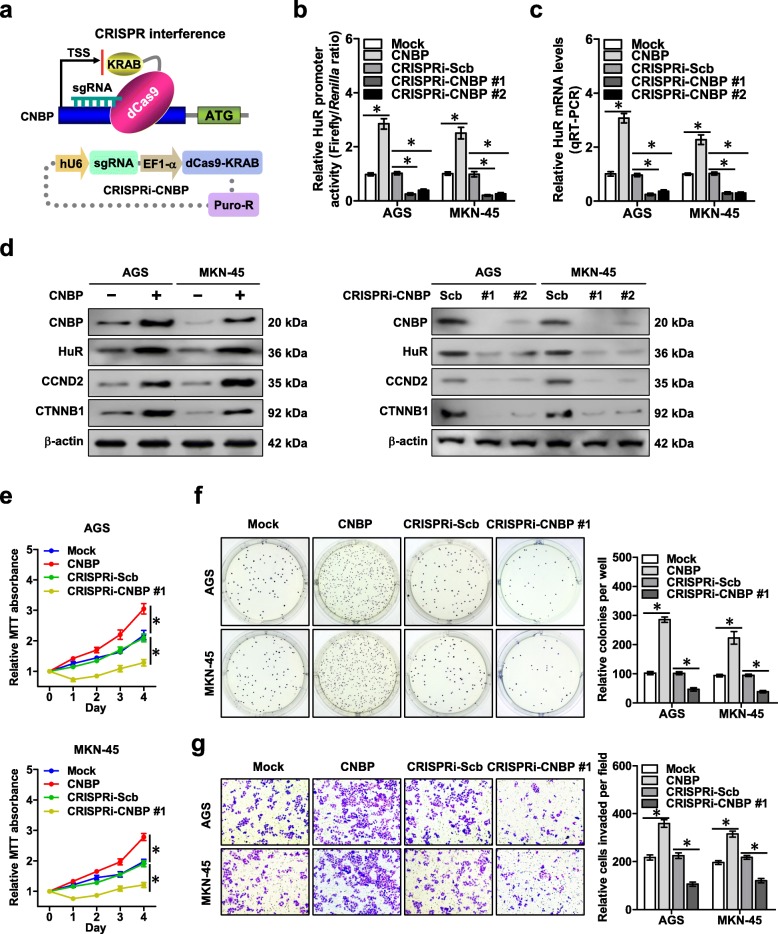
CNBP promotes *HuR* expression, growth, and aggressiveness of gastric cancer. **a** Schematic illustration of dCas9-based CRISPRi for *CNBP* and small guide RNA (sgRNA) targeting region. **b** Dual-luciferase assay revealing the promoter activity of *HuR* in AGS and MKN-45 cells stably transfected with empty vector (mock), *CNBP,* CRISPRi-Scb, CRISPRi-CNBP #1, or CRISPRi-CNBP #2. (**c**) and (**d**) Real-time qRT-PCR (**c,** normalized to β-actin, *n* = 5) and western blot (**d**) assays showing the transcript and protein levels of *HuR* in AGS and MKN-45 cells stably transfected with mock, *CNBP*, CRISPRi-Scb, CRISPRi-CNBP #1, or CRISPRi-CNBP #2. **e** MTT colorimetric assay indicating the viability of AGS and MKN-45 cells stably transfected with mock, *CNBP*, CRISPRi-Scb, or CRISPRi-CNBP #1. **f** and **g** Soft agar (**f**) and matrigel invasion (**g**) assays showing the in vitro growth and invasion of AGS and MKN-45 cells stably transfected with mock, *CNBP*, CRISPRi-Scb, or CRISPRi-CNBP #1. ANOVA analyzed the difference in (**b**, **c** and **e**-**g**). *P < 0.01 vs. mock or CRISPRi-Scb. Data are shown as mean ± SEM (error bars) and representative of three independent experiments in (**b**-**g**)

To investigate the roles of CNBP in gastric cancer progression, dCas9-based Clustered regularly interspaced short palindromic repeats (CRISPR) interference (CRISPRi) [27–30] was applied for *CNBP* knockdown (Fig. 4a). Stable transfection of *CNBP* and two independent gRNAs against *CNBP* (CRISPRi-CNBP) into AGS and MKN-45 cells resulted in increased and reduced levels of *CNBP*, respectively (Additional file 1: Figure S4a). Notably, neither ectopic expression nor knockdown of *circ-HuR* affected the *CNBP* transcript and protein levels in AGS and MKN-45 cells (Additional file 1: Figure S4b, c). ChIP and quantitative PCR (qPCR) indicated endogenous binding of CNBP to *HuR* promoter, which was increased and decreased by over-expression or knockdown of *CNBP*, respectively (Additional file 1: Figure S4d). Stable ectopic expression or knockdown of *CNBP* promoted and attenuated the promoter activity of *HuR* in AGS and MKN-45 cells, respectively (Fig. 4b). Furthermore, the transcript and protein levels of *HuR* and its downstream genes *CCND2* and *CTNNB1* were increased and decreased in AGS and MKN-45 cells with stable over-expression or knockdown of *CNBP*, respectively (Fig. 4c, d and Additional file 1: Figure S4e). In MTT colorimetric, soft agar, and matrigel invasion assays, stable over-expression or knockdown of *CNBP* facilitated and reduced the viability, growth and invasiveness of AGS and MKN-45 cells, respectively (Fig. 4e–g). Mining of a well-defined gastric cancer dataset derived from Kaplan-Meier Plotter (http://kmplot.com/analysis) revealed that higher *CNBP* (*P* = 1.9 × 10^− 3^ and *P* = 5.4 × 10^− 4^) or *HuR* (*P* = 8.5 × 10^− 5^ and *P* = 4.0 × 10^− 5^) expression was associated with lower overall survival (OS) and first progression (FP) survival probability of patients (Additional file 1: Figure S4f). These results reveled that CNBP promoted the *HuR* expression and tumor progression in gastric cancer.

### *Circ-HuR* suppresses *HuR* expression, growth, and invasion of gastric cancer cells via repressing CNBP transactivation

We further investigated the interplay effects between *circ-HuR* and CNBP in regulating *HuR* expression and gastric cancer progression. In a cohort of 81 gastric cancer cases, lower *circ-HuR* expression (*P* = 3.0 × 10^− 3^) and higher expression of *CNBP* (*P* = 7.0 × 10^− 4^) or *HuR* (*P* = 3.5 × 10^− 2^) was associated with lower survival probability of patients (Additional file 1: Figure S4 g). In addition, the *circ-HuR* (*R* = -0.602, *P* < 1.0 × 10^− 4^) or *CNBP* (*R* = 0.683, *P* < 1.0 × 10^− 4^) levels were negatively and positively correlated with those of *HuR* in these gastric cancer tissues, respectively (Additional file 1: Figure S4h). Cox regression analysis revealed that distant metastasis [hazard ratio (*HR*) = 2.809, *P* = 0.002], tumor-node-metastasis (TNM) stage (*HR* = 2.519, *P* = 0.015), *circ-HuR* levels (*HR* = 0.616, *P* = 0.012), and CNBP expression (*HR* = 2.643, *P* = 0.003) were prognostic factors for gastric cancer patients (Additional file 1: Table S1).

**Fig. 5:**
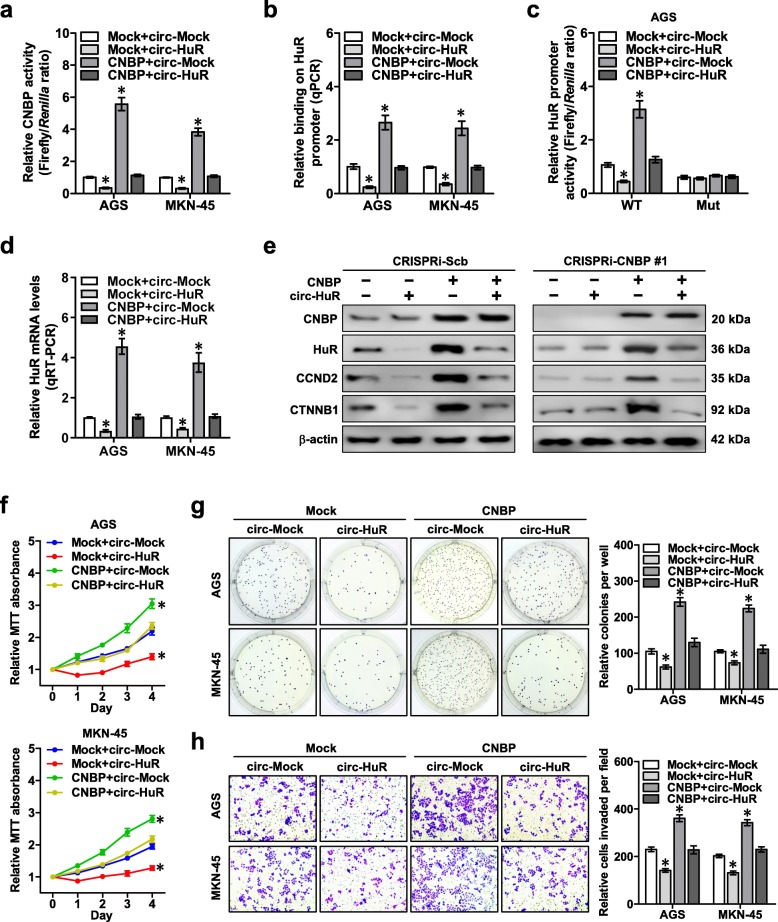
*Circ-HuR* suppresses *HuR* expression, growth, and invasion of gastric cancer cells via repressing CNBP transactivation. **a** Dual-luciferase assay revealing the transactivation of CNBP in AGS and MKN-45 cells stably transfected with empty vector (mock) or *CNBP*, and those co-transfected with *circ-Mock* or *circ-HuR*. **b** ChIP and qPCR assays showing the changes in binding of CNBP to *HuR* promoter in AGS and MKN-45 cells stably transfected with mock or *CNBP*, and those co-transfected with *circ-Mock* or *circ-HuR*. **c** and **d** Dual-luciferase (**c**) and real-time qRT-PCR (**d**) assays indicating the activity of *HuR* promoter with wild type (WT) or mutant (Mut) CNBP binding site and transcript levels (normalized to β-actin, *n* = 4) of *HuR* in AGS and MKN-45 cells stably transfected with mock or *CNBP*, and those co-transfected with *circ-Mock* or *circ-HuR*. **e** Western blot assay showing the expression of CNBP, HuR, CCND2, and CTNNB1 in AGS cells stably transfected with CRISPRi-Scb or CRISPRi-CNBP #1, and those co-transfected with mock, *CNBP*, *circ-Mock*, or *circ-HuR*. **f** MTT colorimetric assay indicating the viability of AGS and MKN-45 cells stably transfected with mock or *CNBP*, and those co-transfected with *circ-Mock* or *circ-HuR*. **g** and **h** Soft agar (**g**) and matrigel invasion (**h**) assays showing in vitro growth and invasion of AGS and MKN-45 cells stably transfected with mock or *CNBP*, and those co-transfected with *circ-Mock* or *circ-HuR*. ANOVA analyzed the difference in (**a**-**d** and **f**-**h**). *P < 0.01 vs. mock+ circ-Mock. Data are shown as mean ± SEM (error bars) and representative of three independent experiments in (**a**-**h**)

In dual-luciferase assay with a reporter containing four canonical CNBP binding sites, ectopic expression or knockdown of *CNBP* facilitated and attenuated the transactivation of CNBP in AGS and MKN-45 cells, respectively (Additional file 1: Figure S5a), while stable over-expression or knockdown of *circ-HuR* suppressed and increased the CNBP transactivation in these cells (Additional file 1: Figure S5b). Notably, ectopic expression of *circ-HuR* attenuated the increase of CNBP transactivation, CNBP enrichment, promoter activity and expression levels of *HuR* induced by over-expression of *CNBP* in AGS and MKN-45 cells (Fig. 5a-e and Additional file 1: Figure S5c). However, neither CNBP nor *circ-HuR* affected the activity of *HuR* promoter containing mutant CNBP binding site (Fig. 5c and Additional file 1: Figure S5c). Ectopic expression of *circ-HuR* did not affect the *HuR* levels in AGS cells stably transfected with CRISPRi-CNBP #1 (Fig. 5e). To further investigate the effects of *circ-HuR* on CNBP transactivation, two established CNBP target genes *MMP-14* [31] and *c-Myc* [32] were chosen for studies. As shown in Additional file 1: Figure S5d-g, stable ectopic expression of *CNBP* facilitated the CNBP enrichment, promoter activity, transcripts and protein levels of *MMP-14* and *c-Myc* in AGS and MKN-45 cells, which were prevented by over-expression of *circ-HuR*. Consistently, stable knockdown of *circ-HuR* increased the CNBP enrichment, promoter activity, transcripts and protein expression levels of *HuR*, *MMP-14* and *c-Myc* in these gastric cancer cells, which were rescued by CRISPRi-mediated knockdown of *CNBP* (Fig. S5 h-k). Importantly, stable *circ-HuR* over-expression attenuated the increase in viability, growth, and invasiveness of AGS and MKN-45 cells induced by ectopic expression of *CNBP* (Fig. 5f–h). These results showed that *circ-HuR* suppressed *HuR* expression, growth, and invasion of gastric cancer cells via repressing CNBP transactivation.

### *Circ-HuR* suppresses gastric cancer progression by inhibiting CNBP transactivation in vivo

**Fig. 6:**
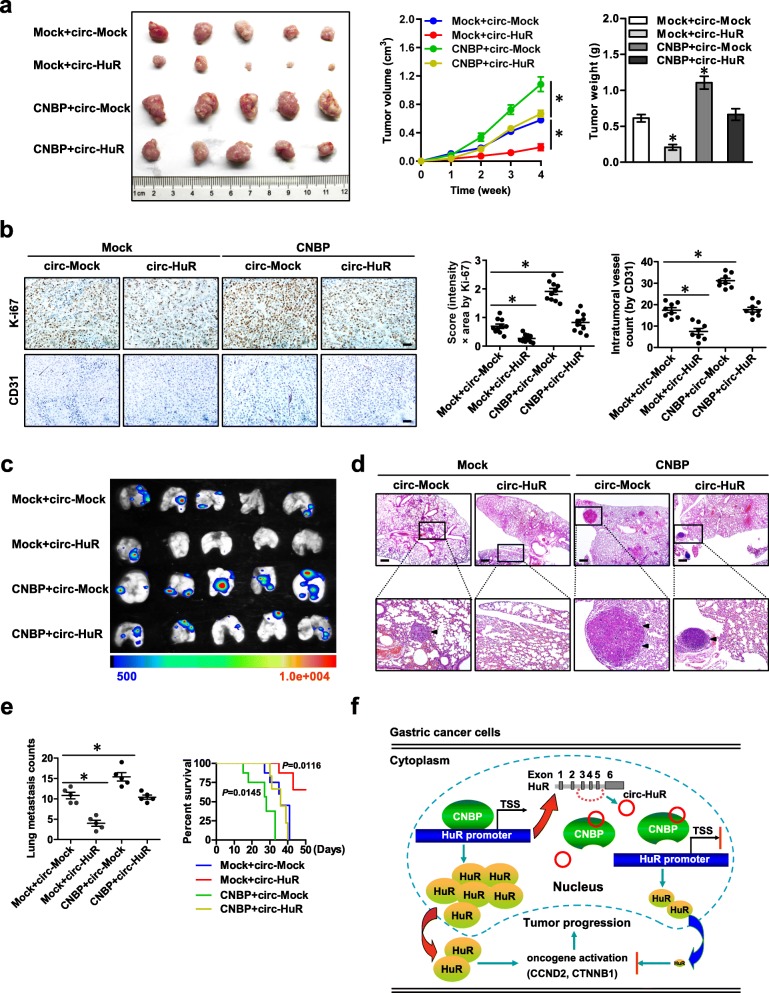
*Circ-HuR* suppresses gastric cancer progression by inhibiting CNBP transactivation in vivo. **a** Representative (left panel), in vivo growth curve (middle panel), and weight at the end points (right panel) of xenograft tumors formed by subcutaneous injection of AGS cells stably transfected with empty vector (mock) or *CNBP*, and those co-transfected with *circ-Mock* or *circ-HuR* into the dorsal flanks of nude mice (*n* = 5 for each group). **b** Representative images (left panel) and quantification (right panel) of immunohistochemical staining showing the expression of Ki-67 and CD31 within xenograft tumors formed by hypodermic injection of AGS cells stably transfected with mock or *CNBP*, and those co-transfected with *circ-Mock* or *circ-HuR* (*n* = 5 for each group). Scale bars: 50 μm. **c**-**e** Representative images (**c**), H&E staining (**d**, arrowheads), and quantification (**e**, left panel) of lung metastatic colonization and Kaplan-Meier curves (**e**, right panel) of nude mice treated with tail vein injection of AGS cells stably transfected with mock or *CNBP*, and those co-transfected with *circ-Mock* or *circ-HuR* (*n* = 5 for each group). Scale bar: 100 μm. **f** The mechanisms underlying *circ-HuR*-suppressed tumor progression: as a nuclear circRNA, *circ-HuR* interacts with CNBP to inhibit its binding to *HuR* promoter, resulting in down-regulation of *HuR* and suppression of gastric cancer progression. ANOVA analyzed the difference in (**a**, **b**, **e**). Log-rank test for survival comparison in (**e**). *P < 0.01 vs. mock+ circ-Mock. Data are shown as mean ± SEM (error bars)

To further confirm the in vitro findings, we observed the biological roles of *circ-HuR* and CNBP in vivo. Consistently, stable transfection of *CNBP* resulted in an obvious increase in the growth and weight of subcutaneous xenograft tumors formed by AGS cells in nude mice, which were attenuated by ectopic expression of *circ-HuR* (Fig. 6a). Immunohistochemical staining revealed the increase of Ki-67 proliferation index and CD31-positive microvessels in xenograft tumors formed by AGS cells stably over-expressing *CNBP*, which were prevented by ectopic expression of *circ-HuR* (Fig. 6b). Importantly, athymic nude mice treated with tail vein injection of AGS cells stably transfected with *CNBP* displayed more lung metastatic colonies and poorer overall survival probability, while stable over-expression of *circ-HuR* attenuated these effects (Fig. 6c-e). Collectively, these results indicated that *circ-HuR* suppressed gastric cancer progression by inhibiting CNBP transactivation in vivo.

## Discussion

With the development of genomic analysis platform, a large number of abundant and conserved circRNAs have been identified in human tissues and cells [33–35]. Recent studies suggest that circRNAs are able to function as tumor drivers or tumor suppressors in multiply ways [10, 14, 16, 36–39]. Besides action mode of binding miRNA, circRNAs participate in parental gene expression at transcriptional level [16, 40]. In this study, we discover that *circ-HuR* (*hsa_circ_0049027*), a novel nuclear circRNA, is down-regulated in gastric cancer. Over-expression of *circ-HuR* suppresses the growth and aggressiveness of gastric cancer in vitro and in vivo. Mechanistically, *circ-HuR* inhibits the enrichment of CNBP on the promoter of *HuR*, resulting in reduction of *HuR* expression (Fig. 6f). These findings highlight a novel tumor suppressive circRNA in regulating tumor growth and aggressiveness, presenting a promising therapeutic target for gastric cancer.

CNBP is a highly conserved zinc finger protein consisting of seven tandem repeats of CCHC zinc finger and an arginine/glycine-rich region, and contributes to embryonic development, organogenesis, and tumorigenesis [26, 31, 41, 42]. As a transcription factor, CNBP binds to the promoters of target genes such as macrophage colony-stimulating factor (*CSF1*) [43], *MMP-14* [31], and *c-Myc* [32]. CNBP expression is elevated in human tumor tissues, and is associated with proliferation, invasion, and migration of tumor cells in vitro and in vivo [31, 44]. In the current study, mining of public gastric cancer datasets reveals that higher expression of *CNBP* is associated with poor outcome of patients. Gain- and loss-of-function studies show that CNBP facilitates *HuR* expression, growth, and aggressiveness of cancer cells, suggesting the oncogenic roles of CNBP in gastric cancer progression. Additionally, our results indicate that RGG box domain is essential for the transcriptional activity of CNBP. We identify that *circ-HuR* is able to suppress the transcriptional activity of CNBP, and functions as an endogenous inhibitor for repressing the binding of CNBP to *HuR* promoter, resulting in down-regulation of *HuR* and its target genes involved in cancer progression.

HuR protein is able to regulate the expression of labile mRNAs containing AU-rich elements through increasing their half-life [45]. As a RBP shuttling between the nucleus and cytoplasm, HuR exerts a fundamental role in tumor progression, and its cytoplasmic presence is intimately linked to mRNA stabilizing function [5, 46]. Many HuR target genes are necessary for cell growth and proliferation, such as *cyclin A*, *cyclin B1*, *cyclin D2* [22, 47], hypoxia-inducible factor 1α (*HIF-1α*), vascular endothelial growth factor (*VEGF*), cyclooxygenase-2 (*COX-2*), and *β-catenin* [8, 23, 48]. Thus, it has been a promising tumor therapeutic approach via modulating the expression or activity of HuR. For example, treatment with MS-444, an inhibitor interfering the RNA binding and trafficking of HuR, results in loss of viability and induction of apoptosis in malignant glioma cells [49]. Additionally, CMLD-2, a disruptor of interaction between HuR and mRNA targets, exerts antitumor effects in thyroid cancer cells by decreasing cell viability and increasing apoptosis [50], highlighting HuR as a promising therapeutic target for cancers. In this study, our evidence shows that *circ-HuR* is able to inhibit *HuR* expression and suppress the growth and aggressiveness of gastric cancer in vitro and in vivo, suggesting a potential therapeutic approach for cancers.

## Conclusions

This study indicates that a novel nuclear circRNA, termed as *circ-HuR*, is down-expressed in gastric cancer tissues and cells. *Circ-HuR* acts as an inhibitor of CNBP transactivation by directly interacting with its RGG domain. Interestingly, CNBP promotes the expression of *HuR* at transcription level via binding to *HuR* promoter in gastric cancer cells. Meanwhile, *circ-HuR* restrains the transcription of *HuR* by inhibiting CNBP transactivation, and suppresses the growth and aggressiveness of gastric cancer in vitro and in vivo. This study extends our knowledge about the regulation of *HuR* expression and gastric cancer progression by circRNA, and provides a potential target for treatment of gastric cancer.

## Supplementary information

**Additional file 1: Figure S1.** Expression of *HuR*-derived circRNAs and their effects on *HuR* expression in gastric cancer cells. **Figure S2.** Effects of *circ-HuR* on expression of *HuR* and downstream genes in gastric cancer cells. **Figure S3.** Interaction between *circ-HuR* and CNBP protein in gastric cancer cells. **Figure S4**. Roles of *circ-HuR* and CNBP in regulating transcription of *HuR* and downstream genes. **Figure S5**. *circ-HuR* regulates CNBP transactivation and target gene expression in gastric cancer cells. **Table S1.** Univariate and multivariate analysis of prognostic factors in gastric cancer patients. **Table S2.** Primer sets used for RT-PCR, qPCR, RIP, and ChIP. **Table S3.** Primer sets used for constructs. **Table S4.** Oligonucleotide sets used for CRISPR-dCas9, short hairpin RNAs, or probe.

**Additional file 2: Table S5.** Over-lapping analysis of *circ-HuR*-binding protein with RNA binding protein and transcription factor.

## Data Availability

The data supporting the conclusions of this article are presented within the article and its Additional files.

## Abbreviations

ATG: Translational start codon
bHLH: Basic helix-loop-helix
CCND2: Cyclin D2
circRNAs: Circular RNAs
c-Myc: MYC proto-oncogene
CNBP: CCHC-type zinc finger nucleic acid binding protein
CRISPR: Clustered regularly interspaced short palindromic repeats
CTNNB1: Catenin beta 1
dCas9: Dead mutant of Cas9 endonuclease
EF1-α: Elongation factor-1α promoter
ENO1: Enolase 1
GAPDH: Glyceraldehyde-3-phosphate dehydrogenase
HDGF: Heparin binding growth factor
hU6: human U6 promoter
HuR: Human antigen R
MMP-14: Matrix metallopeptidase 14
NAP1L1: Nucleosome assembly protein 1 like 1
NONO: Non-POU domain containing octamer binding
Puro-R: Puromycin resistance
qRT-PCR: Quantitative real-time PCR
RIP: RNA immunoprecipitation
RNA-FISH: RNA fluorescence in situ hybridization
RT-PCR: Reverse transcription PCR
SFPQ: Splicing factor proline and glutamine rich
sgRNA: Small guide RNA
shRNA: Short hairpin RNA
sh-Scb: Scramble shRNA
TSS: Transcriptional start site
