# Correction: *Circ-HuR* suppresses HuR expression and gastric cancer progression by inhibiting CNBP transactivation

**DOI:** 10.1186/s12943-023-01862-3

**Published:** 2023-09-20

**Authors:** Feng Yang, Anpei Hu, Dan Li, Jianqun Wang, Yanhua Guo, Yang Liu, Hongjun Li, Yajun Chen, Xiaojing Wang, Kai Huang, Liduan Zheng, Qiangsong Tong

**Affiliations:** 1 Department of Pediatric Surgery, Union Hospital, Tongji Medical College, Huazhong University of Science and Technology, 1277 Jiefang Avenue, Wuhan, Hubei Province 430022 People’s Republic of China; 2 Department of Pathology, Union Hospital, Tongji Medical College, Huazhong University of Science and Technology, 1277 Jiefang Avenue, Wuhan, Hubei Province 430022 People’s Republic of China; 3 Clinical Center of Human Genomic Research, Union Hospital, Tongji Medical College, Huazhong University of Science and Technology, 1277 Jiefang Avenue, Wuhan, Hubei Province 430022 People’s Republic of China

**Correction: Mol Cancer 18, 158 (2019)**

**https://doi.org/10.1186/s12943-019-1094-z**

Following publication of the original article [1], the authors identified errors in Fig. 2f and in Fig. 5h. In Fig. 5h, the transwell invasion images for “AGS Mock + circ-Mock”, “AGS Mock + circ-HuR”, and “MKN-45 Mock + circ-Mock” were misused. After a self-investigation and carefully check of the archived images of this study, the authors found these errors happened inadvertently during the preparation of figures. The correct figures are given below.

**Fig. 2:**
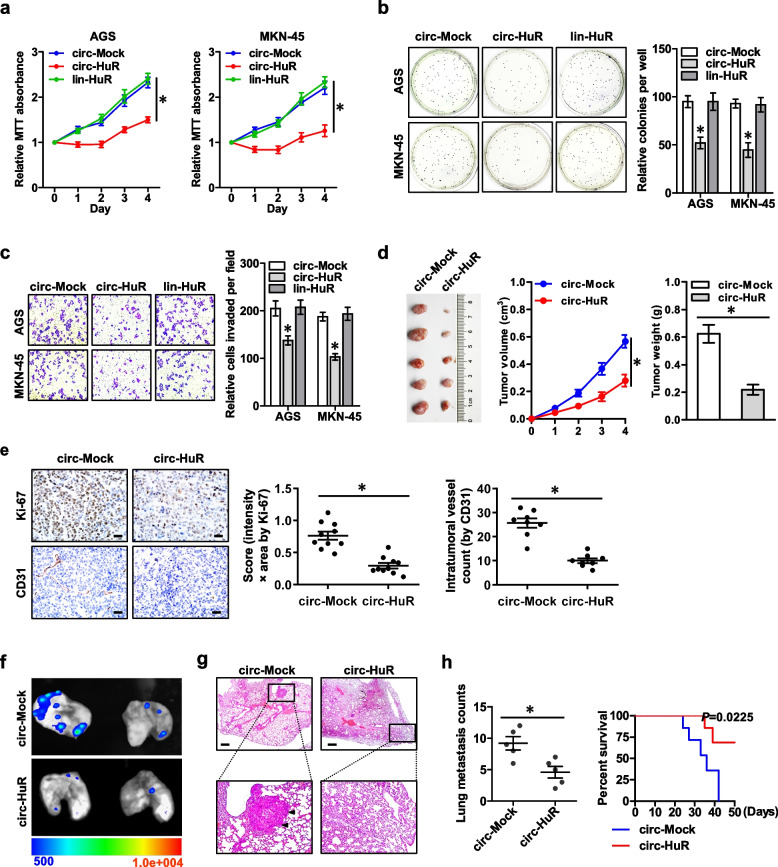
Over-expression of *circ-HuR* suppresses the growth and aggressiveness of gastric cancer. **a** MTT colorimetric assay showing the viability of AGS and MKN-45 cells stably transfected with empty vector (*circ-Mock*), *circ-HuR*, or *lin-HuR*. **b** and **c** Soft agar (**b**) and matrigel invasion (**c**) assays indicating the in vitro growth and invasion of AGS and MKN-45 cells stably transfected with *circ-Mock*, *circ-HuR*, or *lin-HuR*. **d** Representative (left panel), in vivo growth curve (middle panel), and weight at the end points (right panel) of xenograft tumors formed by subcutaneous injection of AGS cells stably transfected with *circ-Mock* or *circ-HuR* into the dorsal flanks of nude mice (*n* = 5 for each group). **e** Representative images (left panel) and quantification (right panel) of immunohistochemical staining showing the expression of Ki-67 and CD31 within xenograft tumors formed by hypodermic injection of AGS cells stably transfected with *circ-Mock* or *circ-HuR* (*n* = 5 for each group). Scale bars: 50 μm. **f**–**h** Representative images (**f**), H&E staining (**g**, arrowheads), and quantification (**h**, left panel) of lung metastatic colonization and Kaplan–Meier curves (**h**, right panel) of nude mice treated with tail vein injection of AGS cells stably transfected with mock or *circ-HuR* (*n* = 5 for each group). Scale bar: 100 μm. ANOVA and Student’s t-test analyzed the difference in **a**-**e** and **h**. Log-rank test for survival comparison in (**h**). *P < 0.01 vs. *circ-Mock*. Data are shown as mean ± SEM (error bars) and representative of three independent experiments in (**a**-**c**)

**Fig. 5:**
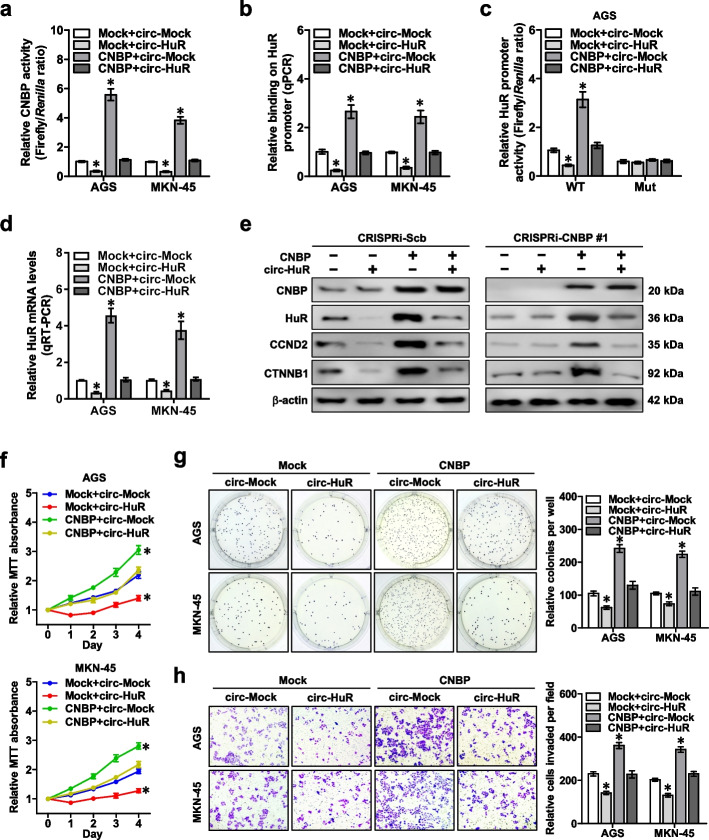
*Circ-HuR* suppresses *HuR* expression, growth, and invasion of gastric cancer cells via repressing CNBP transactivation. **a** Dual-luciferase assay revealing the transactivation of CNBP in AGS and MKN-45 cells stably transfected with empty vector (mock) or CNBP, and those co-transfected with *circ-Mock* or *circ-HuR*. **b** ChIP and qPCR assays showing the changes in binding of CNBP to *HuR* promoter in AGS and MKN-45 cells stably transfected with mock or CNBP, and those co-transfected with *circ-Mock* or *circ-HuR*. **c** and **d** Dual-luciferase (**c**) and real-time qRT-PCR (**d**) assays indicating the activity of *HuR* promoter with wild type (WT) or mutant (Mut) CNBP binding site and transcript levels (normalized to β-actin, *n* = 4) of *HuR* in AGS and MKN-45 cells stably transfected with mock or CNBP, and those co-transfected with *circ-Mock* or *circ-HuR*. **e** Western blot assay showing the expression of CNBP, HuR, CCND2, and CTNNB1 in AGS cells stably transfected with CRISPRi-Scb or CRISPRi-CNBP #1, and those cotransfected with mock, *CNBP*, *circ-Mock*, or *circ-HuR*. **f** MTT colorimetric assay indicating the viability of AGS and MKN-45 cells stably transfected with mock or *CNBP*, and those co-transfected with *circ-Mock* or *circ-HuR*. **g** and **h** Soft agar (**g**) and matrigel invasion (**h**) assays showing in vitro growth and invasion of AGS and MKN-45 cells stably transfected with mock or *CNBP*, and those co-transfected with *circ-Mock* or *circ-HuR*. ANOVA analyzed the difference in (**a**-**d** and **f**–**h**). *P < 0.01 vs. mock + circ-Mock. Data are shown as mean ± SEM (error bars) and representative of three independent experiments in (**a**-**h**)
