# Supplementary material for: Circ-HuR suppresses HuR expression and gastric cancer progression by inhibiting CNBP transactivation
Source: Mol Cancer. 2019 Nov 13;18:158. doi: 10.1186/s12943-019-1094-z (PMC6852727; doi:10.1186/s12943-019-1094-z)
Supplement: Supplementary file 1 — Additional file 1: Figure S1. Expression of HuR-derived circRNAs and their effects on HuR expression in gastric cancer cells. Figure S2. Effects of circ-HuR on expression of HuR and downstream genes in gastric cancer cells. Figure S3. Interaction between circ-HuR and CNBP protein in gastric cancer cells. Figure S4. Roles of circ-HuR and CNBP in regulating transcription of HuR and downstream genes. Figure S5. circ-HuR regulates CNBP transactivation and target gene expression in gastric cancer cells. Table S1. Univariate and multivariate analysis of prognostic factors in gastric cancer patients. Table S2. Primer sets used for RT-PCR, qPCR, RIP, and ChIP. Table S3. Primer sets used for constructs. Table S4. Oligonucleotide sets used for CRISPR-dCas9, short hairpin RNAs, or probe. [file 12943_2019_1094_MOESM1_ESM.pdf]

Additional files

***Circ-HuR* suppresses HuR expression and gastric cancer progression by inhibiting CNBP transactivation**

Yang, et al.

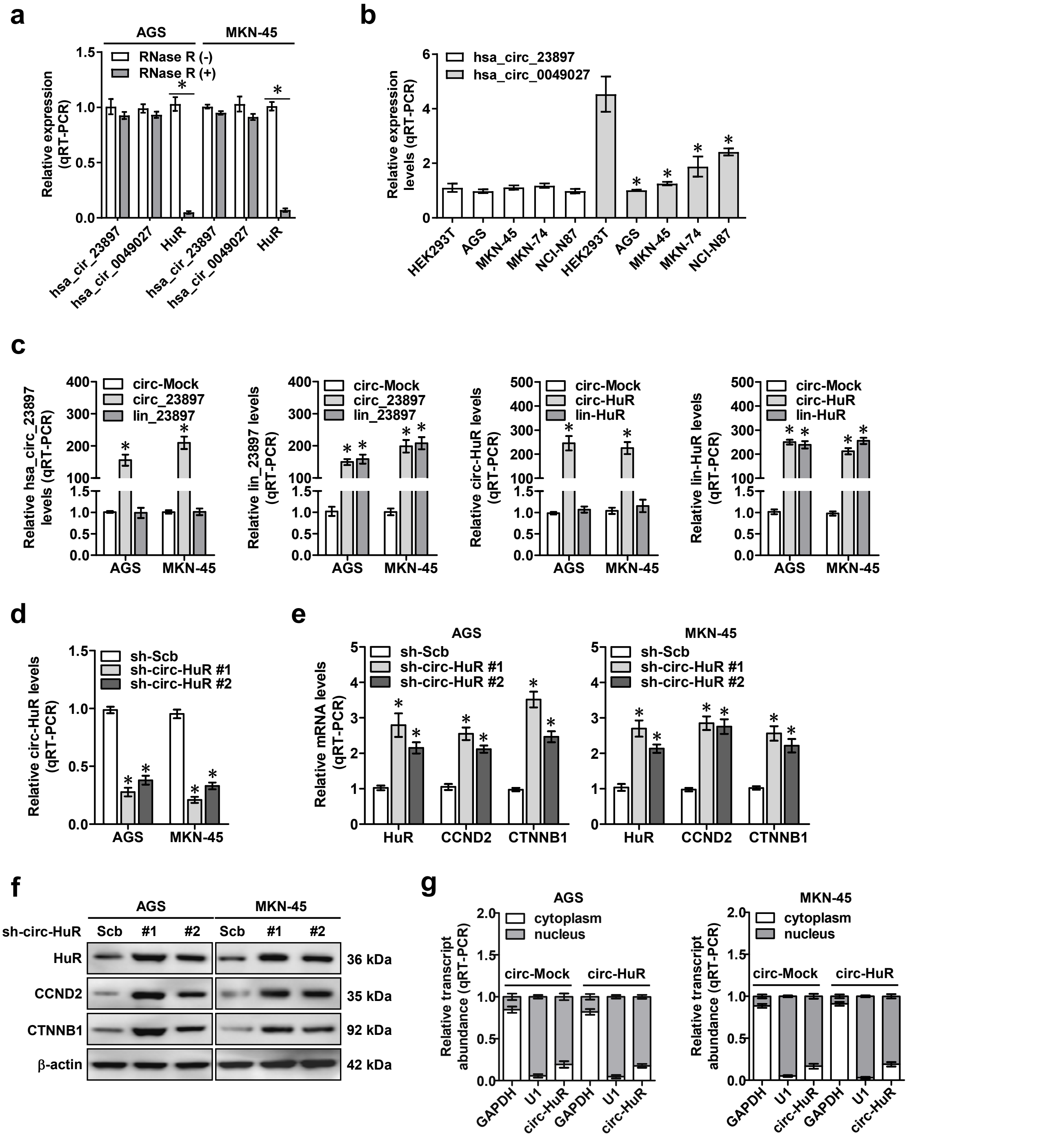

Figure S1. Expression of *HuR*-derived circRNAs and their effects on *HuR* expression in gastric cancer cells. a Real-time qRT-PCR assay indicating the levels (normalized to β-actin, *n*=5) of *hsa_circ_23897* or *hsa_circ_0049027* after RNase R treatment (3 U/mg) in AGS and MKN-45 cells. **b** Real-time qRT-PCR assay revealing the relative levels of *circ-HuR* (normalized to β-actin, *n*=5) in HEK293T and gastric cancer cell lines. **c** Real-time qRT-PCR assay showing the levels (normalized to β-actin, *n*=5) of *hsa_circ-23897*, linear *hsa_circ-23897* (*lin_23897*), *circ-HuR*, and linear *circ-HuR* (*lin-HuR*) in AGS and MKN-45 cells stably transfected with empty vector (*circ-Mock*), *hsa_circ-23897* (*circ_23897*), *lin_23897*, *circ-HuR*, or *lin-HuR*. **d** Real-time qRT-PCR assay indicating the levels (normalized to β-actin, *n*=5) of *HuR* in AGS and MKN-45 cells stably transfected with scramble shRNA (sh-Scb), sh-circ-HuR #1, or sh-circ-HuR #2. **e** and **f** Real-time qRT-PCR (**e**, normalized to β-actin, *n*=5) and western blot (**f**) assays revealing the transcript and protein levels of *HuR* and its downstream target genes in AGS and MKN-45 cells stably transfected with sh-Scb, sh-circ-HuR #1, or sh-circ-HuR #2. **g** Real-time qRT-PCR indicating the distribution of *GAPDH*, *U1*, and *circ-HuR* in the cytoplasmic and nuclear fractions of AGS and MKN-45 cells stably transfected with *circ-Mock* or *circ-HuR*. Student's *t*-test or ANOVA analyzed the difference in **a**-**e**. * $P<0.01$ vs. RNase R (-), HEK293T, circ-Mock, or sh-Scb. Data are shown as mean ±SEM (error bars) and representative of three independent experiments in **a**-**g**.

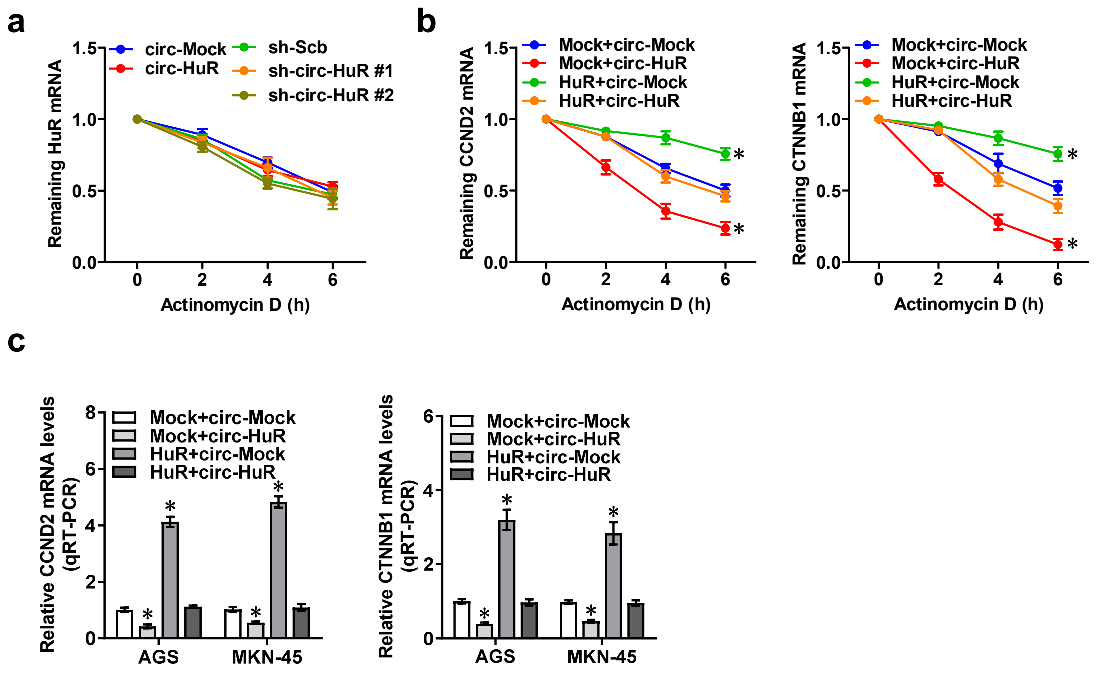

Figure S2. Effects of *circ-HuR* on expression of *HuR* and downstream genes in gastric cancer cells. a Real-time qRT-PCR assay indicating the half-life of *HuR* mRNA (normalized to β-actin, n=5) in AGS cells stably transfected with empty vector (*circ-Mock*), *circ-HuR*, scramble shRNA (sh-Scb), sh-circ-HuR #1, or sh-circ-HuR #2, and those treated with actinomycin D (1 μg/mL) for indicated periods of time. **b** and **c** Real-time qRT-PCR assay showing the half-life and levels of *CCND2* and *CTNNB1* mRNA (normalized to β-actin, n=5) in AGS cells stably transfected with empty vector (mock), *HuR*, *circ-Mock*, or *circ-HuR*, and those treated with actinomycin D (1 μg/mL) for indicated periods of time. Student's t-test or ANOVA analyzed the difference in **a**-**c**. * P<0.01 vs. mock+circ-Mock. Data are shown as mean ±SEM (error bars) and representative of three independent experiments in **a**-**c**.

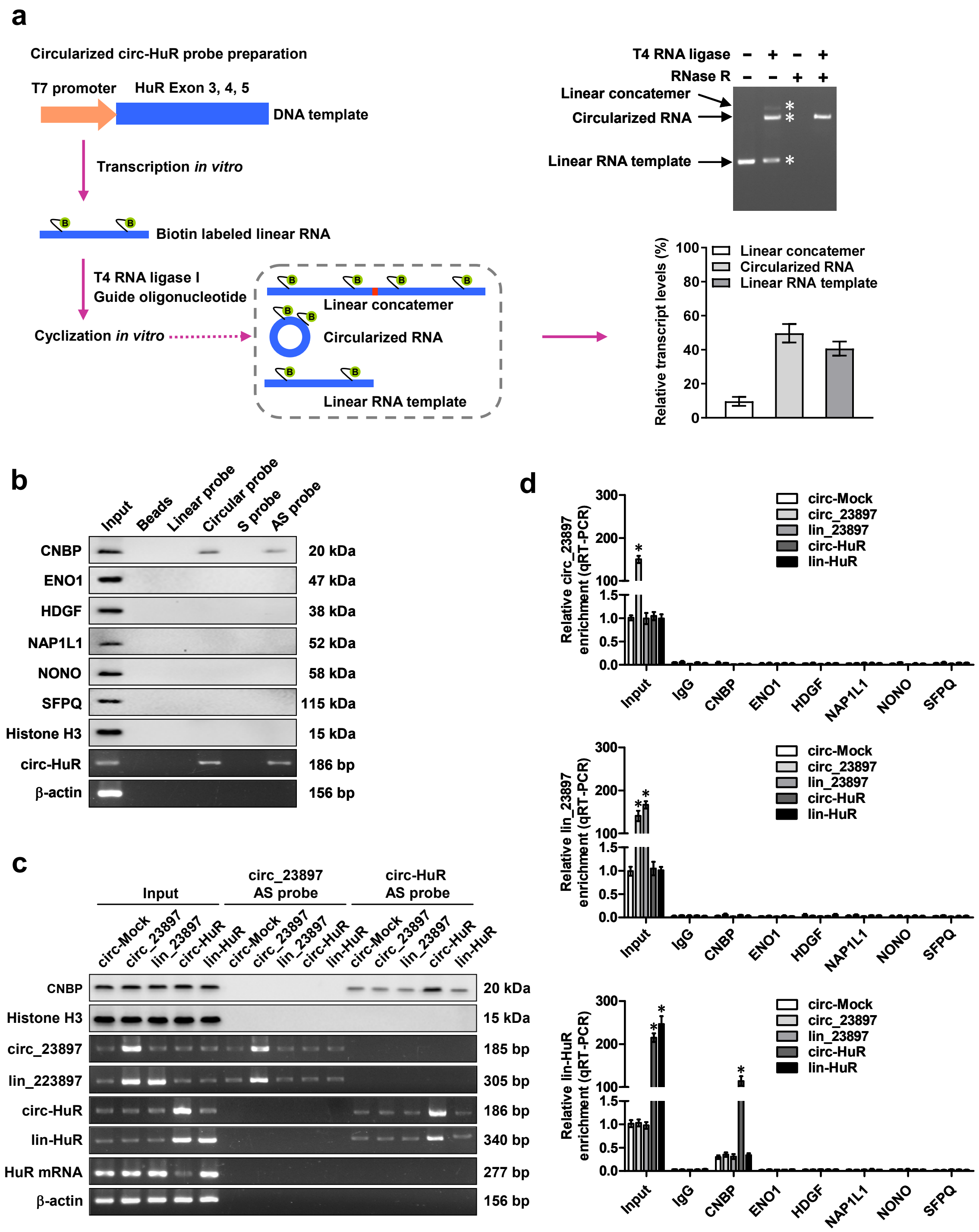

Figure S3. Interaction between *circ-HuR* and CNBP protein in gastric cancer cells. a Schematic diagram showing the process to generate biotin-labeled *circ-HuR* probe by ligation of *in vitro* transcribed linear transcript (left panel), and validation by denaturing PAGE, SYBR Green II staining, and RNase R (3 U/mg) treatment (right upper panel), with circularization efficiency as indicated (right lower panel). **b** Western blot (upper panel) and RT-PCR (lower panel) assays indicating the proteins and transcripts pulled down by biotin-labeled linear or circular exogenous *circ-HUR*, sense (S) or antisense (AS) probe targeting junction site of endogenous *circ-HuR* from AGS cell lysates. **c** Western blot (upper panel) and RT-PCR (lower panel) assays revealing the proteins and transcripts pulled down by biotin-labeled antisense (AS) probe targeting junction site of *hsa_circ-23897* (*circ_23897*) or *circ-HuR* from lysates of AGS cells stably transfected with empty vector (*circ-Mock*), *circ_23897*, linear *circ_23897* (*lin_23897*), *circ-HuR*, or linear *circ-HuR* (*lin-HuR*). **d** RIP and real-time qRT-PCR assays showing the interaction of *circ_23897*, *lin_23897*, or *lin-HuR* with six proteins in AGS cells stably transfected with *circ-Mock*, *circ_23897*, *lin_23897*, *circ-HuR*, or *lin-HuR*. ANOVA analyzed the difference in **d**. * $P<0.01$ vs. circ-Mock. Data are shown as mean±SEM (error bars) and representative of three independent experiments in **b**-**d**.

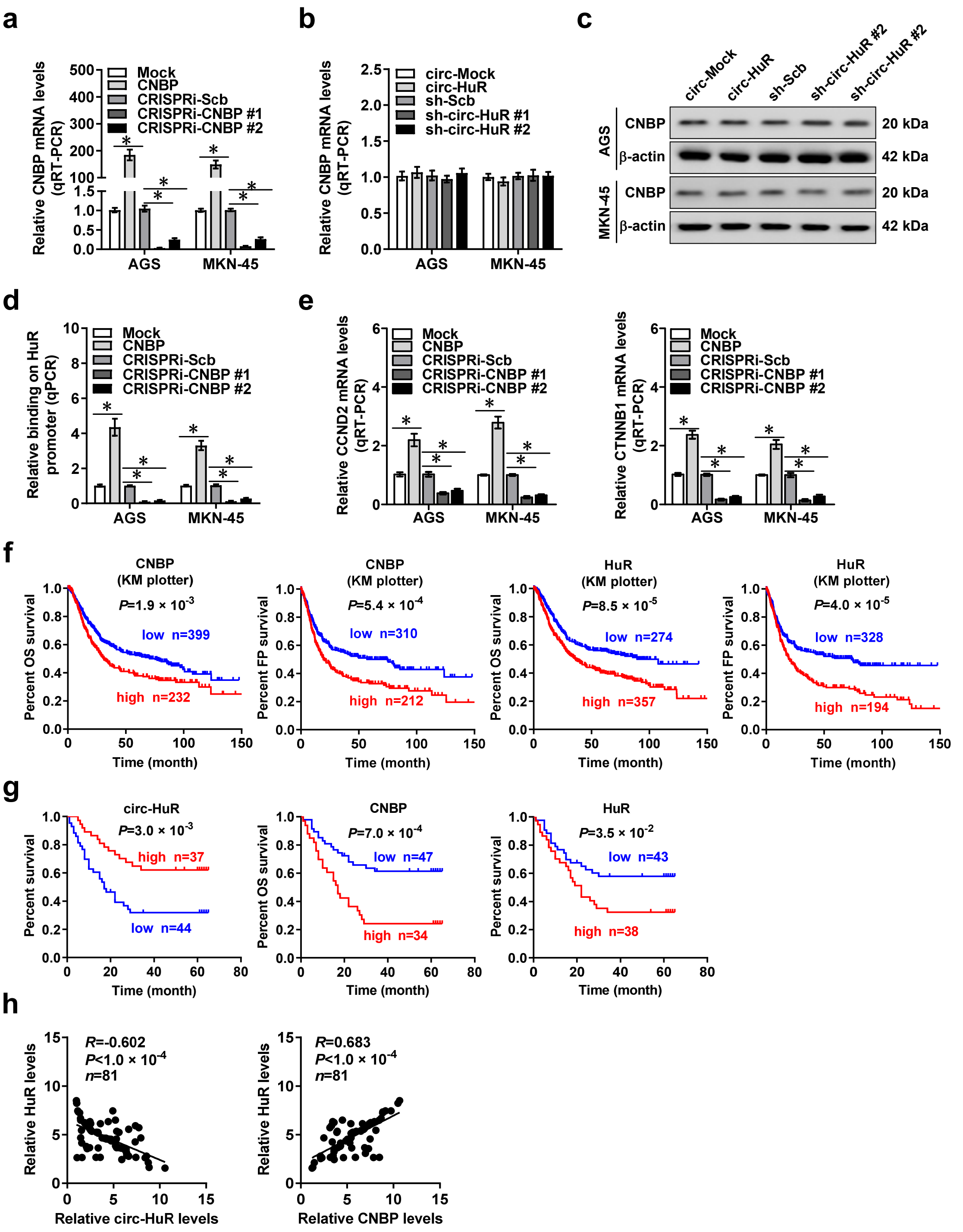

Figure S4. Roles of *circ-HuR* and CNBP in regulating transcription of *HuR* and downstream genes. a Real-time qRT-PCR assay indicating the *CNBP* transcript levels (normalized to β-actin, n=5) in AGS and MKN-45 cells stably transfected with empty vector (mock), *CNBP*, CRISPRi-Scb, CRISPRi-CNBP #1, or CRISPRi-CNBP #2. **b** and **c** Real-time qRT-PCR assay (**b**, normalized to β-actin, n=5) and western blot (**c**) assays showing the transcript and protein levels of *CNBP* in AGS and MKN-45 cells stably transfected with empty vector (*circ-Mock*), *circ-HuR*, sh-Scb, sh-circ-HuR #1, or sh-circ-HuR #2. **d** ChIP and qPCR assays indicating the changes in the binding of CNBP (normalized to input) to *HuR* promoter in AGS and MKN-45 cells stably transfected with mock, *CNBP*, CRISPRi-Scb, CRISPRi-CNBP #1, or CRISPRi-CNBP #2. **e** Real-time qRT-PCR assay revealing the transcript levels (normalized to β-actin, n=5) of *CCND2* and *CTNNB1* in AGS and MKN-45 cells stably transfected with mock, *CNBP*, CRISPRi-Scb, CRISPRi-CNBP #1, or CRISPRi-CNBP #2. **f** Overall (OS) and first progression (FP) survival of gastric cancer samples derived from Kaplan-Meier Plotter database (http://kmplot.com/analysis), with high or low expression of *CNBP* (cutoff values=487.0 and 482.0) or *HuR* (cutoff values=363.5 and 419.5). **g** Kaplan–Meier curves showing the survival of 81 gastric cancer patients with low or high expression of *circ-HuR* (cutoff value=4.1), *CNBP* (cutoff value=5.5), or *HuR* (cutoff value=4.7). **h** The expression correlation of *HuR* with *circ-HuR* or *CNBP* in 81 gastric cancer tissues. Student's t-test or ANOVA analyzed the difference in **a**, **b**, **d**, and **e**. Log-rank test for survival comparison in **f** and **g**. Pearson's correlation coefficient analysis for gene expression in **h**. *P<0.01 vs. mock, CRISPRi-Scb, circ-Mock, or sh-Scb. Data are shown as mean ±SEM (error bars) and representative of three independent experiments in **a**-**e**.

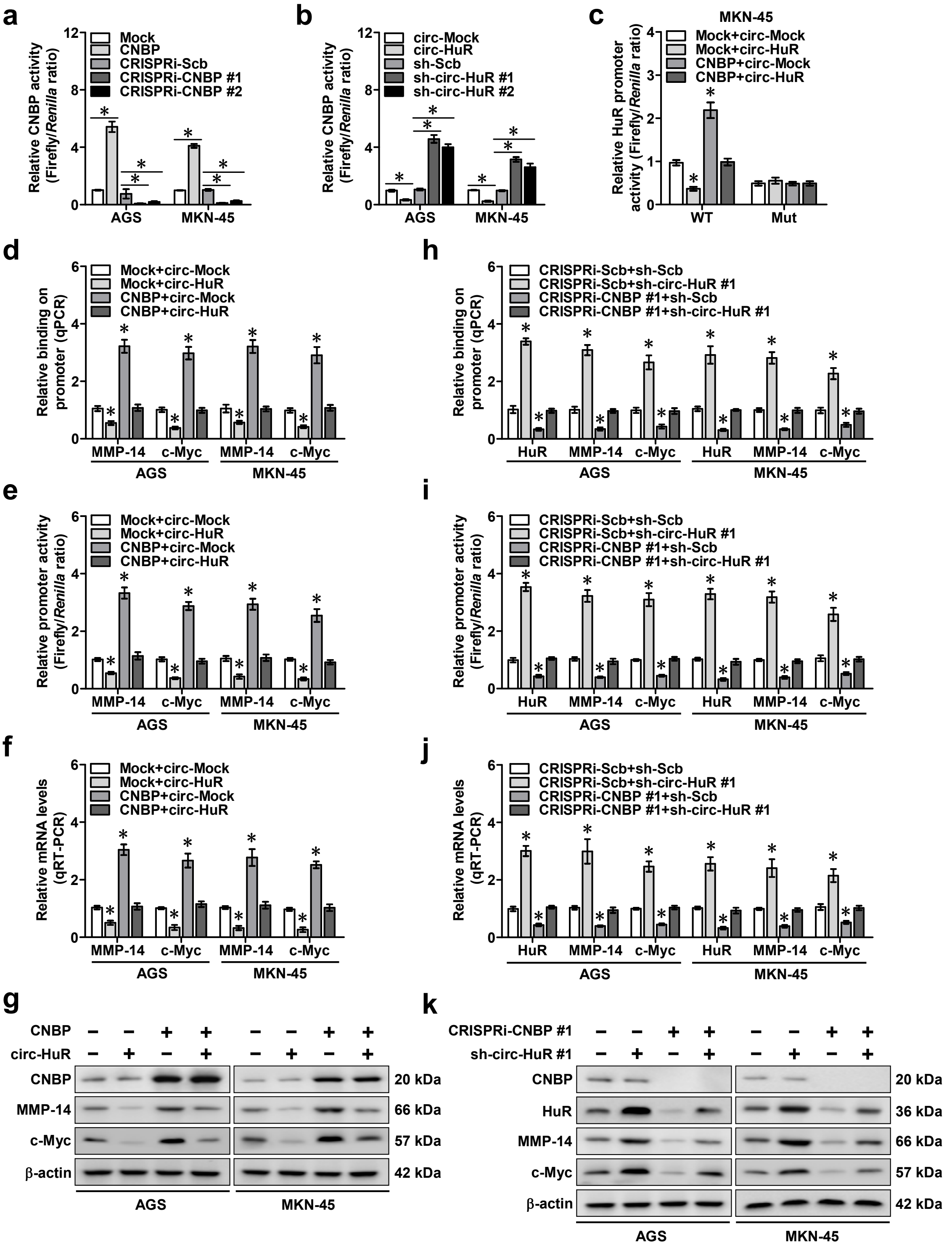

Figure S5. ***circ-HuR* regulates CNBP transactivation and target gene expression in gastric cancer cells. a** Dual-luciferase assay revealing the activity of CNBP in AGS and MKN-45 cells stably transfected with empty vector (mock), *CNBP*, CRISPRi-Scb, CRISPRi-CNBP #1, or CRISPRi-CNBP #2 (*n*=5). **b** Dual-luciferase assay indicating the activity of CNBP in AGS and MKN-45 cells stably transfected with empty vector (*circ-Mock*), *circ-HuR*, scramble shRNA (sh-Scb), sh-circ-HuR #1, or sh-circ-HuR #2 (*n*=6). **c** Dual-luciferase assay showing the activity of *HuR* promoter with wild type (WT) or mutant (Mut) CNBP binding site in AGS and MKN-45 cells stably transfected with mock or *CNBP*, and those co-transfected with *circ-HuR* (*n*=5). **d-g** ChIP and qPCR (d), dual-luciferase reporter (e), real-time qRT-PCR (f, normalized to β-actin, *n*=5), and western blot (g) assays showing the CNBP enrichment, promoter activity, transcript and protein levels of *MMP-14* and *c-Myc* in AGS and MKN-45 cells stably transfected with *circ-Mock* or *circ-HuR*, and those co-transfected with *CNBP*. **h-k** ChIP and qPCR (h), dual-luciferase reporter (i), real-time qRT-PCR (j, normalized to β-actin, *n*=5), and western blot (k) assays showing the CNBP enrichment, promoter activity, transcript and protein levels of *MMP-14* and *c-Myc* in AGS and MKN-45 cells stably transfected with sh-Scb or sh-circ-HuR #1, and those co-transfected with CRISPRi-Scb or CRISPRi-CNBP #1. Student's *t*-test or ANOVA analyzed the difference in **a-f** and **h-j**. * $P<0.01$ vs. mock, CRISPRi-Scb, circ-Mock, sh-Scb, mock+circ-Mock, or CRISPRi-Scb+sh-Scb. Data are shown as mean ±SEM (error bars) and representative of three independent experiments in **a-k**.

Table S1 Univariate and multivariate analysis of prognostic factors in gastric cancer patients

Clinicopathologic factor	n	Univariate analysis		Multivariate analysis		
		Mean ± SEM (months)	*P*-value	Hazard ratio	95% CI	*P*-value
**Age (years)**						
≤60	42	39.1 ± 4.1	0.332	1.374	0.755-2.500	0.298
>60	39	34.3 ± 4.2				
**Sex**						
Male	57	33.9 ± 3.5	0.168	0.572	0.281-1.163	0.123
Female	24	43.7 ± 5.3				
**Size (diameter)**						
≤6 cm	40	33.6 ± 4.2	0.221	0.736	0.404-1.341	0.736
>6 cm	41	39.9 ± 4.1				
**Laurén classification**						
Intestinal type	47	36.3 ± 3.8	0.731	0.822	0.444-1.521	0.533
Diffuse type	34	37.5 ± 4.6				
**Gastric wall invasion**						
T1/T2	34	49.1 ± 4.1	<0.001	1.266	1.066-2.901	0.213
T3/T4	47	27.9 ± 3.6				
**Lymph node metastasis**						
Negative	22	51.6 ± 5.3	<0.001	1.368	1.052-2.894	0.135
Positive	59	31.2 ± 3.3				
**Distant metastasis**						
Negative	62	41.9 ± 3.2	<0.001	2.809	1.432-5.511	0.002
Positive	19	20.3 ± 5.4				
**TNM stage**						
I/II	27	48.3 ± 4.7	0.003	2.519	1.193-5.319	0.015
III/IV	54	31.1 ± 3.5				
**circ-HuR expression**						
Low	44	28.3 ± 3.8	0.003	0.616	0.440-0.861	0.012
High	37	46.9 ± 3.9				
**CNBP expression**						
Low	47	45.4 ± 3.7	<0.001	2.643	1.194-4.260	0.003
High	34	25.1 ± 4.0				
**HuR expression**						
Low	43	43.0 ± 4.1	0.035	1.411	1.030-1.932	0.232
High	38	29.9 ± 4.0				

HuR, human antigen R; circ-HuR, circular RNA derived from HuR; CNBP, CCHC-type zinc finger nucleic acid binding protein; TNM, tumor-node-metastasis. Log-rank test and Cox regression model were applied for univariate and multivariate analysis. *n*, number of patients; SEM, standard error of the mean; 95% CI, 95% confidence interval.

Table S2 Primer sets used for RT-PCR, qPCR, RIP, and ChIP

Primer set	Primers	Sequence	Product size (bp)	Application
circ-HuR	Forward	5’-ACAAAAACGTGGCACTCCTCT-3'	186	RT-PCR
(Divergent)	Reverse	5’-GGTTTTTGACTGGAGCCTCAA-3'		qPCR, RIP
lin-HuR	Forward	5’-TTGAGGCTCCAGTCAAAAACC-3'	340	RT-PCR
(Convergent)	Reverse	5’-AGAGGAGTGCCACGTTTTTGT-3'		qPCR, RIP
hsa_circ_23897	Forward	5’-ATGACCCAGAAGGACGTAGAAG-3'	185	RT-PCR
(Divergent)	Reverse	5’-CTGAGGGAGGTAGTTGACGATC-3'		qPCR
lin_circ_23897	Forward	5’-GATCGTCAACTACCTCCCTCAG-3’	305	RT-PCR
(Convergent)	Reverse	5’-CTTCTACGTCCTTCTGGGTCAT-3'		qPCR, RIP
hsa_circ_0049026	Forward	5’-CAGGTTCGTGTTAAACGCTGT-3'	180	RT-PCR
(Divergent)	Reverse	5’-GGTTTTTGACTGGAGCCTCAA-3'		qPCR
β-actin	Forward	5'-AAATCGTGCGTGACATTAAGGAGA-3’	-	RT-PCR
(Divergent)	Reverse	5'-CATACCCCTCGTAGATGGGCA-3’		
β-actin	Forward	5'-TGCCCATCTACGAGGGGTATG-3'	156	RT-PCR,
(Convergent)	Reverse	5'-TCTCCTTAATGTCACGCACGATTT-3'		qPCR
GAPDH	Forward	5'-AGAAGGCTGGGGCTCATTTG-3'	258	qPCR
	Reverse	5'-AGGGGCCATCCACAGTCTTC-3'		
U1	Forward	5'-ACTTACCTGGCAGGGGAGATACC-3’	137	qPCR
	Reverse	5'-CCACTACCACAAATTATGCAGTCG-3’		
HuR	Forward	5'-GTCCTCGTGGATCAGACTAC-3'	277	qPCR
	Reverse	5'-TCATGTGATCGACGCCCATG-3’		
CNBP	Forward	5'-GCTACAACTGTGGCAAACC-3'	213	qPCR
	Reverse	5'-GTGCAAGGTGCCCTGACT-3’		
CCND2	Forward	5'-ACCTTCCGCAGTGCTCCTAC-3'	161	qPCR
	Reverse	5'-CCCAGCCAAGAAACGGTCCA-3'		
CTNNB1	Forward	5'-TGCTGAAGGTGCTATCTGTC-3'	198	qPCR
	Reverse	5'-GAAGCTGAACAAGAGTCCCA-3'		
MMP-14	Forward	5’-GCCTTCTGTTCCTGATAA-3'	225	qPCR
	Reverse	5’-CCATCCTTCCTCTCGTAG-3'		
c-Myc	Forward	5'-ATTCTCTGCTCTCCTCGACG-3'	224	qPCR
	Reverse	5'-CAGTGGGCTGTGAGGAGGTT-3'		
HuR ChIP	Forward	5'-TGCATGTATTTCTACTAGGGCATT-3'	225	qPCR
(-679/-455)	Reverse	5'-TGAGGAGGCGGGGCATGGAACG-3'		
MMP-14 ChIP	Forward	5'-CTCCCTTCCCTACATACCTCCA-3'	247	qPCR
(-850/-604)	Reverse	5'-GTCATTTGTAATGTGGCTGCAC-3'		
c-Myc ChIP	Forward	5'-CTCCCGTCTAGCACCTTTGATT-3'	239	qPCR
(-1199/-961)	Reverse	5'-GGCGTCTGTTTAGCCCTGAGAT-3'		

HuR, human antigen R; circ-HuR, circular RNA derived from HuR; GAPDH, glyceraldehyde 3-phosphate dehydrogenase; U1, U1 small nuclear 1; CNBP, CCHC-type zinc finger nucleic acid binding protein; CCND2, cyclin D2; CTNNB1, catenin beta 1; MMP-14, matrix metallopeptidase 14; c-Myc, MYC proto-oncogene, bHLH transcription factor; RT-PCR, reverse transcription PCR; qPCR, quantitative PCR; RIP, RNA immunoprecipitation; ChIP, chromatin immunoprecipitation.

Table S3 Primer sets used for constructs

Primer Set	Sequences
pLCDH-circ-HuR	5'-CCGGAATTCTGAAATATGCTATCTTACAGGACACAGCTTGGGCTATGGCTTT-3' (sense); 5'-CGCGGATCCTCAAGAAAAAATATATTCACCTGAATCTCTGCGCCTGGTGGTG-3' (antisense)
pLCDH-lin-HuR	5'-CCGGAATTCTGAAATATGCTATCTTACAGGACACAGCTTGGGCTATGGCTTT-3' (sense); 5'-CGCGGATCCGGCTGAATCTCTGCGCCTGGTGGTG-3' (antisense)
pLCDH-circ_23897	5'-CCGGAATTCTGAAATATGCTATCTTACAGATTTTTGAAAAATACAATGTCTA-3' (sense); 5'-CGCGGATCCTCAAGAAAAAATATATTCACCTGTAGTCTGATCCACGAGGACC-3' (antisense)
pLCDH-lin_23897	5'-CCGGAATTCTGAAATATGCTATCTTACAGATTTTTGAAAAATACAATGTCTA-3' (sense); 5'-CGCGGATCCGGCTGTAGTCTGATCCACGAGGACC-3' (antisense)
pCMV-3Tag-1A-CNBP-WT	5'-CGCGGATCCATGAGCAGCAATGAGTGCTT-3' (sense); 5'-CCGCTCGAGTTAGGCTGTAGCCTCAATTG-3' (antisense)
pCMV-3Tag-1A-CNBP-ΔRGG-1	5'-CGCGGATCCATGAGCAGCAATGAGTGCTT-3' (sense-1); 5'-CAAACTGGAAAGTAGGACATTCCCGGGCCC-3' (antisense-1)
pCMV-3Tag-1A-CNBP-ΔRGG-2	5'-ATGTCCTACTTTCCAGTTTGTTTCCTCGTC-3' (sense-2); 5'-CCGCTCGAGTTAGGCTGTAGCCTCAATTG-3' (antisense-2)
pCMV-3Tag-1A-CNBP-Δ1-RGG	5'-CGCGGATCCCCAGACATTTGTTATCGCTG-3' (sense); 5'-CCGCTCGAGTTAGGCTGTAGCCTCAATTG-3'' (antisense)
pCMV-3Tag-1A-CNBP-Δ5-7	5'-CGCGGATCCATGAGCAGCAATGAGTGCTT-3' (sense); 5'-CCGCTCGAGTGCATGGTCGCAGTCACGAG-3' (antisense)
pGEX-6P-1-CNBP-WT	5'-CGCGGATCCATGAGCAGCAATGAGTGCTT-3' (sense); 5'-CCGCTCGAGTTAGGCTGTAGCCTCAATTG-3' (antisense)
pGEX-6P-1-CNBP-ΔRGG-1	5'-CGCGGATCCATGAGCAGCAATGAGTGCTT-3' (sense-1); 5'-CAAACTGGAAAGTAGGACATTCCCGGGCCC-3' (antisense-1)
pGEX-6P-1-CNBP-ΔRGG-2	5'-ATGTCCTACTTTCCAGTTTGTTTCCTCGTC-3' (sense-2); 5'-CCGCTCGAGTTAGGCTGTAGCCTCAATTG-3' (antisense-2)
pGEX-6P-1-CNBP-Δ1-RGG	5'-CGCGGATCCCCAGACATTTGTTATCGCTG-3' (sense); 5'-CCGCTCGAGTTAGGCTGTAGCCTCAATTG-3'' (antisense)
pGEX-6P-1-CNBP-Δ5-7	5'-CGCGGATCCATGAGCAGCAATGAGTGCTT-3' (sense); 5'-CCGCTCGAGTGCATGGTCGCAGTCACGAG-3' (antisense)
pGL3-HuR promoter WT	5'-CGGGGTACCGGCACGAGAATCACTTGAATAGGG-3' (sense); 5'-CCCAAGCTTCCGCCCGGTAAGGTAGGAAAGGT-3' (antisense)
pGL3-HuR promoter Mut	5'-TTTAATGAATCCGCACCCTGCTGAGCCTTCGGGAACTGGCTTGCTCAA-3' (sense); 5'-AGGCTCAGCAGGGTGCGGATTCATTAAAATGTCATTGAGCGCCTACTG-3' (antisense)
pGL3-CNBP reporter	5'-CAAATGAGAATAAAATGAGATATCTGAAAATATACTGAAAATA-3' (sense); 5'-AGCTTATTTTCAGTATATTTTCAGATATCTCATTTTATTCTCATTTGGTAC-3' (antisense)
pGL3-c-Myc promoter	5'-CGGAAGATCTGTTTGTCCGGGGAGGAAAGAGTT-3' (sense); 5'-CCCAAGCTTCGCTGGAATTACTACAGCGAGTT-3' (antisense)

HuR, human antigen R; lin, linear; CNBP, CCHC-type zinc finger nucleic acid binding protein; RGG, arginineglycine-glycine-rich region; WT, wild type; Mut, mutation; c-Myc: MYC proto-oncogene, bHLH transcription factor.

Table S4 Oligonucleotide sets used for CRISPR-dCas9, short hairpin RNAs, or probe

Oligo Set		Sequences
sgRNA-CRISPRi-CNBP #1	Forward	5'-CACCGGCCGTGTGCAGACCCGCGTG-3'
	Reverse	5'-AAACCACGCGGGTCTGCACACGGCC-3'
sgRNA-CRISPRi-CNBP #2	Forward	5'-CACCGGCGTGTGGCGCAGGCAAGGA-3'
	Reverse	5'-AAACTCCTTGCCTGCGCCACACGCC-3'
sh-Scb	Antisense	5'-CCGGGCGAACGATCGAGTAAACGGACTCGAGTCCGTTTACTCGATCGTTCGCTTTTT-3'
	Sense	5'-AATTCAAAAAGCGAACGATCGAGTAAACGGACTCGAGTCCGTTTACTCGATCGTTCGC-3'
sh-circ-HuR #1	Antisense	5'-CCGGTCAGAGATTCAGGACACAGCCTCGAGGCTGTGTCCTGAATCTCTGTTTTTG-3'
	Sense	5'-GATCCAAAAACAGAGATTCAGGACACAGCCTCGAGGCTGTGTCCTGAATCTCTGA-3'
sh-circ-HuR #2	Antisense	5'-CCGGTAGATTCAGGACACAGCTTGCTCGAGCAAGCTGTGTCCTGAATCTTTTTTG-3'
	Sense	5'-GATCCAAAAAAGATTCAGGACACAGCTTGCTCGAGCAAGCTGTGTCCTGAATCTA-3'
circ-HuR probe	Antisense	5'-AGCCCAAGCTGTGTCCTGAATCTCTGCGCC-3'
	Sense	5'-GGCGCAGAGATTCAGGACACAGCTTGGGCT-3'
circ_23897 probe	Antisense	5'-GTATTTTTCAAAAATCTGTAGTCTGATCCACGAGG-3'
HuR mRNA probe	Antisense	5'-ATCGACGCCCATGGGGGAGAACCTGAATCT-3'
circ-HuR transcription *in vitro*	Forward	5'-TAATACGACTCACTATAGGGGACACAGCTTGGGCTATGGCTTT-3'
	Reverse	5'-CTGAATCTCTGCGCCTGGTGGTG-3'
circ-HuR circularization	Guide DNA	5'-AGCCCAAGCTGTGTCAATCTCTGCGCCTGG-3'

sgRNA, small guide RNA; CRISPRi, clustered regularly interspaced short palindromic repeats interference; dCas9, dead mutant of Cas9 endonuclease; Scb, scramble; shRNA, short hairpin RNA; HuR, human antigen R; circ-HuR, circular RNA derived from HuR.
